# Proteomic profiling of *Pseudomonas aeruginosa *AES-1R, PAO1 and PA14 reveals potential virulence determinants associated with a transmissible cystic fibrosis-associated strain

**DOI:** 10.1186/1471-2180-12-16

**Published:** 2012-01-22

**Authors:** Nathan J Hare, Nestor Solis, Christopher Harmer, N Bishara Marzook, Barbara Rose, Colin Harbour, Ben Crossett, Jim Manos, Stuart J Cordwell

**Affiliations:** 1 School of Molecular Bioscience, The University of Sydney, Sydney 2006, Australia; 2 Discipline of Immunology and Infectious Diseases, School of Medical Sciences, The University of Sydney, Sydney 2006, Australia; 3 Discipline of Pathology, School of Medical Sciences, The University of Sydney, Sydney 2006, Australia; 4 School of Molecular Bioscience, Building GO8, Maze Crescent, The University of Sydney, Sydney 2006, Australia

## Abstract

**Background:**

*Pseudomonas aeruginosa *is an opportunistic pathogen that is the major cause of morbidity and mortality in patients with cystic fibrosis (CF). While most CF patients are thought to acquire *P. aeruginosa *from the environment, person-person transmissible strains have been identified in CF clinics worldwide. The molecular basis for transmissibility and colonization of the CF lung remains poorly understood.

**Results:**

A dual proteomics approach consisting of gel-based and gel-free comparisons were undertaken to analyse protein profiles in a transmissible, early (acute) isolate of the Australian epidemic strain 1 (AES-1R), the virulent burns/wound isolate PA14, and the poorly virulent, laboratory-associated strain PAO1. Over 1700 *P. aeruginosa *proteins were confidently identified. AES-1R protein profiles revealed elevated abundance of proteins associated with virulence and siderophore biosynthesis and acquisition, antibiotic resistance and lipopolysaccharide and fatty acid biosynthesis. The most abundant protein in AES-1R was confirmed as a previously hypothetical protein with sequence similarity to carbohydrate-binding proteins and database search revealed this gene is only found in the CF-associated strain PA2192. The link with CF infection may suggest that transmissible strains have acquired an ability to rapidly interact with host mucosal glycoproteins.

**Conclusions:**

Our data suggest that AES-1R expresses higher levels of proteins, such as those involved in antibiotic resistance, iron acquisition and virulence that may provide a competitive advantage during early infection in the CF lung. Identification of novel proteins associated with transmissibility and acute infection may aid in deciphering new strategies for intervention to limit *P. aeruginosa *infections in CF patients.

## Background

*Pseudomonas aeruginosa *is a Gram negative opportunistic pathogen with an extraordinary capacity to survive in, and adapt to, a wide range of environmental niches. Genome size (approximately 5500 genes [1]) and plasticity enable the expression of an arsenal of surface-associated and secreted virulence factors [2], which contribute to nosocomially-acquired *P. aeruginosa *infections, particularly those involving burns and wounds, as well as meningitis, endocarditis and microbial keratitis. *P. aeruginosa *is also the major determinant of morbidity and mortality in patients suffering from the autosomal recessive disorder cystic fibrosis (CF) [3]. CF patients display electrolyte transport imbalance and dysfunction across many organ systems. In the lungs, this is characterized by the production of a thickened dehydrated mucus layer, which provides an environment suitable for colonization by pathogens [4]. Although many species are able to colonize the CF lung, including *Staphylococcus aureus *and *Haemophilus influenzae*, *P. aeruginosa *will eventually dominate in the majority of patients. Initial *P. aeruginosa *infections may be cleared by antibiotics, however biofilm formation allows persistence that is associated with antibiotic resistance and chronic infection [5]. Strains of *P. aeruginosa *associated with CF infections are likely to contain and/or express genes that confer functional traits allowing initial colonization of the CF lung mucosa as well as the ability to out-compete other pathogens.

Contrary to the dogma that CF patients acquire unique *P. aeruginosa *from an environmental source [6], it has now become evident that person-to-person transmissible strains may circulate within CF clinics [7-11]. Such strains have been found in the United Kingdom and Europe (Manchester epidemic strain [MA], Liverpool epidemic strain [LES] [10,11] and Clone C [12]), as well as Canada [13] and Australia (Australian epidemic strain 1 [AES-1] [7]). Increasing evidence suggests that transmission between patients occurs via a cough-associated aerosol route [14,15]. The majority of epidemic strains display evidence of increased virulence in CF patients [16] and transmission to patients with non-CF bronchiectasis, or even otherwise healthy relatives, has been detected [17]. Little is known however, about the mechanisms underlying transmissibility and pathogenesis of epidemic *P. aeruginosa*. Isolates from initial infection tend to be non-mucoid and motile, but over time the organism undergoes genotypic and phenotypic changes that promote persistence, including conversion to mucoidy, loss of motility and reduced type III secretion consistent with biofilm formation [18]. Whole genome sequencing of two clonally related isolates collected from a CF patient 7.5 years apart [18] (early infection and chronic infection) showed loss of function in virulence genes required for O-antigen biosynthesis, type III secretion, twitching motility, exotoxin A regulation, multi-drug efflux, phenazine biosynthesis, quorum sensing (QS) and iron acquisition.

Horizontal gene transfer and recombination in gene islands, large chromosomal inversions and gene loss are important in *P. aeruginosa *evolution [19,20], and phenotypic traits may also be acquired from infecting bacteriophage. *P. aeruginosa *Clone C carries a plasmid and genomic islands with sequences substantially different from the *P. aeruginosa *reference clone PAO1 that may confer enhanced colonization and survival [21]. Adaptation by *P. aeruginosa *to the CF lung is also accelerated by the host immune response and nutrient limitation, including oxidative stress and iron availability, as well as antibiotic challenge. While the Clone C, AES-1, LES and MA epidemic strains do not appear closely related to each other [22], a shared phenotype is the expression of biofilm-associated genes. Analysis of CF isolates show increased expression of QS, bacteriophage and other genes that are indicative of iron limited, stationary phase, and oxygen-limited growth [23,24] and many of these correlate with in vivo transcriptome analysis [25]. Despite the accumulation of evidence regarding gene expression during infection, the molecular basis for transmissibility is almost completely unknown.

In this study, we employed a complementary proteomic approach involving two-dimensional gel electrophoresis (2-DE) and two-dimensional liquid chromatography coupled to tandem mass spectrometry (2-DLC-MS/MS) with isobaric tags for relative and absolute quantitation (iTRAQ) to determine protein abundance differences between the reference strain *P. aeruginosa *PAO1, the virulent burn/wound isolate UCBPP-PA14 (PA14) and the early, transmissible CF-associated *P. aeruginosa *AES-1R. We identified over 1700 proteins of which 183 were present at statistically significant altered abundance between strains. This study identified 3 previously hypothetical proteins only expressed in strain AES-1R, of which AES_7139 was the most abundant protein detected on 2-DE gels. Other proteins present at elevated abundance in AES-1R compared to PA14 and PAO1 included several secreted and iron acquisition proteins, such as those associated with pyochelin synthesis and binding. AES-1R displayed an absence or decreased abundance of a number of porins including OprE, OprG and OprD, but elevated abundance of the multi-drug efflux protein MexX, part of the MexXY-OprM tripartite efflux pump. AES-1R also displayed differential abundance of proteins involved in lipopolysaccharide and fatty acid biosynthesis. These data suggest that AES-1R expresses specific proteins and regulates the abundance of proteins shared with other *P. aeruginosa *strains to influence transmissibility and colonization of the CF lung.

## Methods

### Bacterial strains and growth conditions

*P. aeruginosa *PAO1 is a laboratory reference strain originally isolated from an infected burn/wound of a patient in Melbourne, Australia (American Type Culture Collection ATCC 15692), strain PA14 (UBPPC-PA14) was obtained from Dr. Laurence Rahme, Harvard Medical School, Cambridge, MA [26] and AES-1R was obtained from Prof. David Armstrong, Monash Medical Centre, Australia [7]. Strains were cultured in six replicates of 50 mL of salt modified Luria-Bertani broth (5 g/L NaCl) and grown to stationary phase (OD_600 nm _~ 1.0) with incubation at 37°C and shaking at 250 × rpm (Additional file 1). Cultures were harvested, washed three times with phosphate-buffered saline and cells collected by centrifugation at 6,000 × *g *for 10 mins at 4°C. The resulting bacterial cell pellets were frozen, lyophilized and stored at -80°C.

### Phenotypic assays

Phenotypic assays on *P. aeruginosa *AES-1R, PAO1 and PA14 were conducted using standard methodologies. Briefly, mucoidy (from - [non-mucoid] to +++ [highly mucoid]) and colony size were assessed by growth on Columbia horse blood agar (Oxoid, Basingstoke UK) and Mueller-Hinton (Oxoid) agar. Pyocyanin production was scored against colour standards from overnight LB broth cultures grown at 37°C. For pyoverdine production, 5 μL of overnight LB culture was spotted on to a King's B agar plate, allowed to dry and incubated for 24 h at 37°C, and assayed based on the zone of pigmentation around the colony. Rhamnolipid, phospholipase C (PLC), haemolysin, total protease and elastase assays were conducted using 5 μL each of overnight LB culture spotted onto agar as follows: i) rhamnolipid, M9 agar; ii) PLC, egg yolk agar (Oxoid); iii) haemolysin, Columbia horse blood agar; iv) total protease, 10 mL skim milk agar; and v) elastase, 10 mL elastin agar. Each assay was incubated for 24-48 h at 37°C and the diameters of clearing zones measured. Each assay was conducted in at least triplicate. Biofilm forming properties were measured using a 1:100 dilution of an overnight LB broth culture in fresh LB medium. 100 μL was added to each well of a flat bottom MicroTest tissue culture plate (BD, Franklin Lakes NJ) and incubated in a moist environment at 37°C for 24 h. Wells were stained with 200 μL 0.5% crystal violet for 3 h before dissolving in 200 μL 20% (v/v) acetone. Absorbance was then read at 620 nm. Swimming motility was assayed by spotting a single colony onto a 0.3% LB agar plate and incubating for 24 h at 37°C. Twitching motility was assayed by stabbing a colony into the bottom of a 10 mL 1% LB agar plate and incubating for 24 h at 37°C. In both cases motility was measured by the diameter of the resulting growth zones.

### Preparation of protein extracts for 2-DE

Proteins were extracted from 10 mg of lyophilized bacterial cell pellets in 1 mL 40 mM Tris (pH 7.8) by tip-probe sonication (Branson, Danbury CT) using 4 cycles of 30 s with resting on ice between cycles. Nucleic acids were removed by incubation with 150 U endonuclease (Sigma, St. Louis MO) for 20 mins at room temperature. Lysates were then centrifuged at 12,000 × *g *for 15 mins at 15°C to remove insoluble material. Resulting supernatants were methanol precipitated overnight at -80°C and the proteins collected by centrifugation at 12,000 × *g *for 30 mins at 4°C. Proteins were then resuspended in 1 mL of 2-DE buffer (5 M urea, 2 M thiourea, 2% [w/v] CHAPS, 2% [w/v] sulfobetaine 3-10, 40 mM Tris, 0.2% [v/v] carrier ampholytes, 0.002% [v/v] bromophenol blue and 2 mM tributylphosphine [TBP]).

### Separation of proteins by 2-DE

Proteins (250 μg) were loaded onto 17 cm pH 4-7 immobilized pH gradient (IPG) strips (Bio-Rad, Hercules CA) by overnight passive rehydration. Isoelectric focussing was carried out using a Bio-Rad IEF Cell for a total of 80 kVh. Proteins in IPG strips were detergent exchanged, reduced and alkylated in 6 M urea, 2% (w/v) SDS, 20% (v/v) glycerol, 5 mM TBP, 2.5% (v/v) acrylamide monomer and 375 mM Tris-HCl (pH 8.8) for 20 mins. Strips were then embedded on an 8-18%T gradient SDS-PAGE gel using 0.5% (w/v) agarose in 25 mM Tris, 192 mM glycine, 0.1% (w/v) SDS. Proteins were separated in a Dodeca Cell (Bio-Rad) at 16°C at 10 V constant voltage for 30 mins followed by 100 V for 16 h. Gels were fixed in 40% (v/v) methanol, 10% (v/v) acetic acid for 1 h and then stained overnight in Sypro Ruby (Bio-Rad). Gels were destained in 10% (v/v) methanol, 7% (v/v) acetic acid for 1 h and imaged using a Molecular Imager Fx (Bio-Rad). Gels were 'double-stained' for a minimum of 24 h in Colloidal Coomassie Blue G-250 (0.1% (w/v) G-250 in 17% (w/v) ammonium sulphate, 34% (v/v) methanol and 3% (v/v) ortho-phosphoric acid). Gels were destained in 1% (v/v) acetic acid for a minimum of 1 h.

Changes in protein abundance were compared for 2-DE gels generated from each strain using the program PD-Quest (Bio-Rad). Since the *x,y*-coordinates of spots on 2-DE gels from different bacterial isolates are not always identical due to minor amino acid sequence variations that lead to altered electrophoretic migration, we undertook a protein mapping exercise to identify like proteins across isolates, as well as image-based comparisons. Spots between isolates corresponding to the same protein identifications were detected using PD-Quest and the relative spot intensities (in ppm) calculated. Statistical analyses were performed on six replicate 2-DE gels corresponding to two gels from each of three separate biological preparations. The cut-off for significance was an *n*-fold change in mean spot abundance of less than 0.67 or greater than 1.5 with a *p*-value less than 0.05, or spots with a ratio less than 0.77 or greater than 1.3 with a *p*-value less than 0.01. Mean spot density values were calculated for each spot across replicate gels and standard error of the mean (SEM) determined. Spots absent from a given strain were denoted as not detected (-), while those only present in that strain were labeled (+). If the SEM was greater than 15% of the calculated mean, the spot was not investigated further. Students' *t*-test was performed on the normalized spot intensities, with significance levels set at 0.05.

### Protein identification by matrix-assisted laser desorption/ionization time-of-flight mass spectrometry (MALDI-TOF MS) peptide mass mapping

Spots were destained in a 60:40 solution of 40 mM NH_4_HCO_3 _(pH 7.8)/100% acetonitrile (MeCN) for 1 h. Gel pieces were vacuum-dried for 1 h and rehydrated in 8 μL of 12 ng/μL of trypsin at 4°C for 1 h. Excess trypsin was removed and gel pieces re-suspended in 25 μL of 40 mM NH_4_HCO_3 _and incubated overnight at 37°C. Peptides were concentrated and desalted using C_18 _Zip-Tips (Millipore, Bedford MA) and eluted in matrix (α-cyano-4-hydroxy cinnamic acid (Sigma), 8 mg/mL in 70% [v/v] MeCN/1% [v/v] formic acid [FA]) directly onto a target plate. Peptide mass maps were generated by matrix-assisted laser desorption/ionisation time-of-flight mass spectrometry (MALDI-TOF MS) using a QSTAR Elite mass spectrometer (Applied Biosystems, Foster City CA) equipped with o-MALDI source. Spectra were examined in Analyst v2.0 (Applied Biosystems) and mass calibration performed prior to data acquisition using external calibration with the Sequazyme™ peptide mass standard kit (Applied Biosystems). Peak lists from each spot were generated by manual interrogation of the spectra. Data from peptide mass maps were used to perform searches of a composite *P. aeruginosa *database composed of translated genome sequences from PAO1 (Pseudomonas Genome Database v2, 2009-11-23), PA14 (Pseudomonas Genome Database v2, 2009-10-14) and AES-1R (unpublished genome sequence data) via an in-house MASCOT server (Matrix Science; v2.2; [complete database 18.694 protein entries]). Identification parameters included peptide mass accuracy within 0.08 Da, one possible missed tryptic cleavage per peptide and with the methionine sulfoxide and cysteine-acrylamide modifications checked. Identifications were based on MASCOT score, observed p*I *and mass (kDa), number of matching peptide masses and total percentage of the amino acid sequence that those peptides covered.

Where insufficient data were obtained for a confident identification using peptide mass mapping, reversed phase liquid chromatography coupled to tandem MS (RPLC-MS/MS) with *de novo *sequencing of peptides was performed. Protein spots were digested as above and the peptides concentrated and desalted using a column packed with Poros R2 resin [27]. Columns were primed with 97% MeCN, acidified with 0.1% trifluoroacetic acid (TFA), and the digested peptides loaded. Bound peptides were washed twice with 0.1% TFA and eluted with 70% MeCN/0.1% TFA. Eluted peptides were dried by vacuum centrifugation and resuspended in 0.1% FA. Peptides were separated using an automated Agilent 1100 nanoflow LC system coupled to an Applied Biosystems Q-STAR Elite mass spectrometer for MS/MS sequencing. Peptides were eluted over 30 mins using a gradient of 5-60% buffer B (0.1% [v/v] FA, 100% MeCN) at a nanoflow rate of 600 nL/min. MS survey scans were performed over the *m/z *range of 400-1800 (three scans), followed by three data-dependent MS/MS scans. Data were analyzed using Analyst and the resulting MS/MS data were searched against the aforementioned *P. aeruginosa *database using MASCOT with the following parameters; allow 1 missed cleavage, precursor mass tolerance 0.2 Da, fragment ion mass tolerance 0.6 Da, with methionine sulfoxide, cysteine-acrylamide, and carbamidomethylation variable modifications selected.

### Quantitative proteomics using iTRAQ and two-dimensional liquid chromatography/tandem mass spectrometry (2-DLC-MS/MS)

Proteins were extracted from 10 mg of lyophilized bacteria in 1 mL 0.1% (w/v) SDS by tip-probe sonication as described above. Proteins were precipitated from the cell lysate by methanol/chloroform as per [28] and collected by centrifugation at 9000 × *g *for 2 mins. The resulting supernatant was removed and the pellet washed 3 times with 1 mL ice-cold methanol. Protein pellets were resuspended in 0.5 M triethylammonium bicarbonate (TEAB; pH 7.8)/0.1% (w/v) SDS. Proteins from each of the *P. aeruginosa *isolates were then labelled with isobaric tags for relative and absolute quantitation (iTRAQ; Applied Biosystems). 50 μg of each protein sample was reduced with 10 mM Tris 2-carboxyethyl phosphine (TCEP) at 60°C for 1 h, then alkylated with 9 mM methyl (methylthio)methyl sulfoxide (MMTS) at room temperature for 10 mins, followed by digestion with trypsin (6 μg/50 μg protein) at 37°C overnight. Digested protein samples were dried by vacuum centrifugation and resuspended in 0.5 M TEAB. Duplicate 4-plex iTRAQ experiments were conducted with the following labelling of samples: *P. aeruginosa *PAO1 (label 114), *P. aeruginosa *PA14 (115), *P. aeruginosa *AES-1R (116), and a biological replicate of *P. aeruginosa *PAO1 (117) in the first experiment and AES-1R in the second. Samples were labelled according to the manufacturer's instructions. Briefly, iTRAQ labels were resuspended in 70 μL ethanol and added to the appropriate protein sample. Labelling was conducted at room temperature for 2 h, and the reaction quenched with 100 μL ultra-pure water. Labelling efficiency was tested by pooling 2 μL aliquots of all labelled samples, desalting peptides as described above, and then acquiring MALDI TOF-TOF MS/MS data on a 4700 mass spectrometer (Applied Biosystems). All samples showed a 1:1:1:1 labelling efficiency. Labelled samples were pooled, dried to near completion by vacuum centrifugation and resuspended in 5 mM phosphate buffer/25% (v/v) MeCN (pH 2.7).

Labelled peptides were then separated by two-dimensional liquid chromatography (2-DLC) and identified by MS/MS. Peptides were fractionated by strong cation exchange (SCX) chromatography using an Agilent 1100 HPLC with a PolyLC (Columbia MD) polysulfoethyl A 200 mm × 2.1 mm 5 μm 200 Å column. Peptides were loaded and washed in buffer A (5 mM phosphate buffer/25% [v/v] MeCN, pH 2.7). Fractions were then collected at 2-4 min intervals during an 80 mins gradient from 10% to 45% buffer B (5 mM phosphate buffer/350 mM KCL/25% [v/v] MeCN, pH 2.7) over 70 mins and then, following a rapid increase, to 100% buffer B for 10 mins at a flow rate of 300 μL/min. SCX fractions were vacuum concentrated and resuspended in 100 μL of 0.1% (v/v) TFA/2% (v/v) MeCN. A Tempo nanoLC (Eksigent, Dublin CA) and Q-Star Elite mass spectrometer (Applied Biosystems) were used for nanoLC electrospray ionisation MS-MS. A 40 μL aliquot of the resuspended sample was loaded on a reverse phase Captrap (Michrom Bioresources, Auburn CA) column and desalted at 10 μL per min for 13 mins. After desalting, the trap was switched on-line and peptides separated by reversed phase chromatography using a 150 μm × 10 cm C18 3 μm 300 Å ProteCol column (SGE Analytical Science, Ringwood Australia). After washing with buffer A (0.1% [v/v] FA), buffer B (90% [v/v] MeCN/0.1% [v/v] FA) concentration was increased from 5% to 90% in 120 mins using three linear gradient steps. The reverse phase nanoLC eluent was subjected to positive ion nanoflow electrospray ionisation MS/MS analysis in an information dependant acquisition mode (IDA). A TOF MS survey scan was acquired (*m/z *380-1600, 0.5 s scan time), with the three most intense multiply charged ions (counts > 50) in the survey scan subjected to MS/MS. MS/MS spectra were accumulated in the mass range *m/z *100-1600 with a modified Enhance All Q2 transition setting favouring low mass ions so that the reporter iTRAQ tag ions (114, 115, 116 and 117 Da) were enhanced for relative quantitation.

Protein identification was performed by combining all the data from each SCX fraction following acquisition of the MS/MS raw files. All data were processed using ProteinPilot (Applied Biosystems, version 2.01) using the Paragon search algorithm. The software correction factors provided in the iTRAQ manufacturer's instructions were entered in the iTRAQ Isotope Correction Factors table. Data were searched using the combined *P. aeruginosa *database described above and a randomized version of the database to calculate false discovery rate (FDR). Search parameters included trypsin as the proteolytic enzyme, up to two possible missed cleavages and MMTS as the selected alkylating agent. Data were filtered to a 1% FDR and the minimum number of unique peptides was set to 1. For all proteins identified on the basis of a single confident peptide identification, the MS/MS spectra were manually verified according to [29]. Spectra with missing iTRAQ labels were omitted from quantitative analysis, unless the corresponding gene was not present in one or more *P. aeruginosa *strains under study. iTRAQ ratios less than 0.67 or greater than 1.5 with a *p*-value less than 0.05, or proteins with a ratio less than 0.77 or greater than 1.3 with a *p*-value less than 0.01, and with consistent results across replicate iTRAQ experiments were deemed significantly differentially abundant.

## Results

### Phenotypic analysis of *P. aeruginosa *AES-1R compared to PAO1 and PA14

*P. aeruginosa *AES-1R was isolated from a child aged 14 months at the same time as the deaths of 5 CF infants infected with AES-1 [7]. The genome sequence has recently been completed [30]. We undertook phenotypic assays to determine the general virulence properties of AES-1R compared to PAO1 and PA14 (Table 1). AES-1R displayed small colonies after 48 h and may therefore qualify as a small colony variant. AES-1R was also non-mucoid and displayed little biofilm formation capability, which are traits consistent with acute CF isolates. For virulence factor assays, AES-1R produced high levels of pyocyanin, and more elastase, total protease and PLC than PAO1 (but less than PA14). Rhamnolipid and hemolysin appeared to be consistent between PAO1 and AES-1R. AES-1R was also comparable in both swimming and twitching motility with both PAO1 and PA14.

**Table 1:** Phenotypic characterization of *P.aeruginosa *AES-1R compared to PAO1 and PA14

Phenotypic Characteristic	AES-1R	PAO1	PA14
Mucoidy (+/-)	-	-	-
Pyocyanin (+/-)	+++	+	+++
Pyoverdine (+/-)	+	+	+
Biofilm (Abs 620 nm)	0.06 ± 0.03	0.11 ± 0.04	0.27 ± 0.06
Elastase (dmm)	17.67 ± 3.12	12.00 ± 0.67	21.33 ± 2.01
Rhamnolipid (dmm)	9.0 ± 0.50	10.0 ± 0.7	11.0 ± 1.0
Phospholipase C (dmm)	17.33 ± 0.87	16.25 ± 1.02	23.33 ± 1.67
Hemolysin (dmm)	7.0 ± 0.4	7.0 ± 0.8	11.0 ± 0.6
Total Protease (dmm)	17.0 ± 1.3	14.0 ± 1.4	19.0 ± 2.3
Swimming Motility (dmm)	37.50 ± 4.79	29.25 ± 5.87	35.00 ± 1.06
Twitching Motility (dmm)	12.5 ± 3.8	17.3 ± 1.1	NP

dmm; diameter in mm; +/-, characteristics measured on a relative scale of (-) no evidence of that phenotype; (+) low, (++) intermediate and (+++) high. NP, not performed

### Comparative gel-based proteomics of *P. aeruginosa *PAO1, PA14 and AES-1R

**Figure 1:**
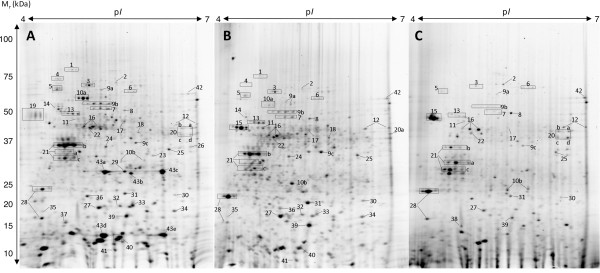
**Two-dimensional gel electrophoresis of proteins from *P. aeruginosa *AES-1R (A), PAO1 (B), and PA14 (C)**. Spot numbers refer to protein identifications as shown in Additional file 2. Boxes indicate positions of multiple spots with the same identification.

Soluble proteins were extracted from stationary phase LB broth cultures of *P. aeruginosa *strains PAO1, PA14 and AES-1R, and separated by 2-DE. All visible protein spots were excised and identified by MALDI-TOF MS peptide mass mapping following in-gel trypsin digestion. Since many potentially 'unique' protein spots detected by image analysis may be accounted for by minor amino acid sequence differences between isolates that result in spot shifts (change in 2-DE *x,y*-coordinates), we performed statistical analysis only on spots with the same identity, or those that were identified in one isolate alone. A total of 154 unique proteins were identified from 563 spots (data not shown), with 54 spots (representing 43 unique proteins) displaying a significant difference in abundance between AES-1R and either, or both of, PAO1 and PA14 (Figure 1 and Additional file 2).

Analysis of the spots that changed in abundance showed that 27 were altered identically (statistically significant change in abundance and either increased or reduced in abundance) in AES-1R compared to both PAO1 and PA14. A further 16 spots were altered in abundance in AES-1R compared to PA14, but not PAO1, while 9 spots were altered in AES-1R compared to PAO1, but not PA14. A single spot (spot 31) was statistically significantly more abundant in AES-1R compared to PA14, but less abundant in AES-1R compared to PAO1, while an additional spot (spot 20d) was present at lower abundance in AES-1R than PA14, but not detected in PAO1. The differentially abundant proteins were functionally clustered into 4 major groups: i) membrane-associated proteins; ii) heat shock proteins/chaperones; iii) oxidative stress proteins; and iv) previously hypothetical proteins. Fourteen proteins were classified as 'membrane-associated', of which 8 (Opr86, PA4595, OprQ, flagellin type B, OprF, PA5217, OmpA and PasP) were elevated in abundance in AES-1R, and 6 (FptA, OmpH, PA2291, OprE, OprD and OprG) were present at lower abundance in AES-1R, compared to either or both, of PAO1 and PA14. All 4 heat shock proteins (HtpG, DnaK, GroEL and PA4352) were elevated in AES-1R compared to both PAO1 and PA14. Five proteins involved in oxidative stress resistance (PA3529, AhpC, PA4880, PA2331 and KatA) were altered in AES-1R, with all except KatA present at increased abundance. Additional smaller functional clusters included the 3 enzymes of the arginine deiminase pathway (ArcABC) and the ATP synthase alpha and beta subunits.

**Figure 2:**
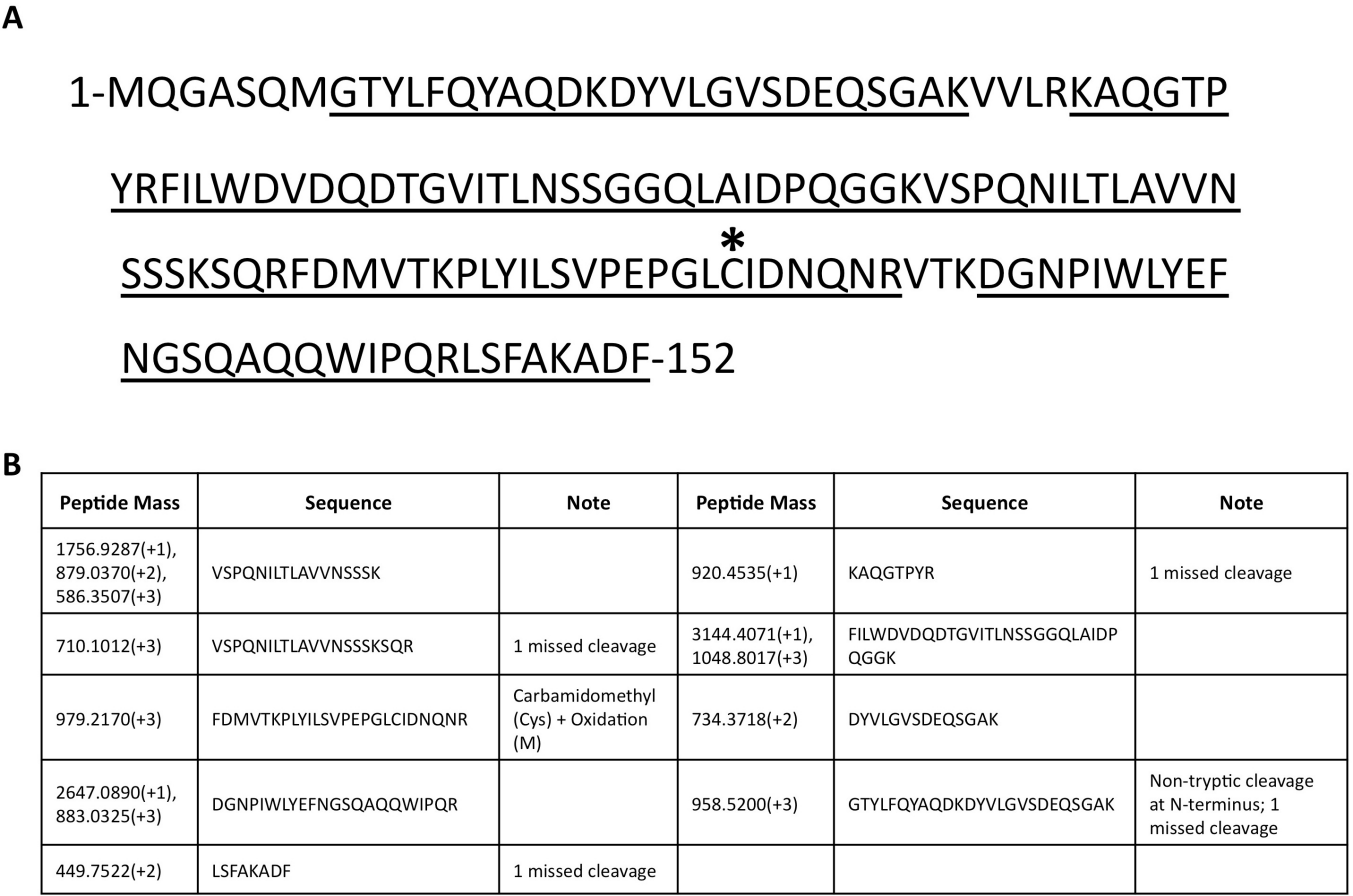
**Predicted protein sequence of a *P. aeruginosa *AES-1R hypothetical protein ((A); AES_7139; spot 43a-e) characterized by MALDI-MS and LC-MS/MS (B)**. Peptide Mass, peptides observed by either LC-MS/MS (charge states > +1) or by peptide mapping (charge state +1); Sequence, peptide sequence defined by assignment of *y*- and *b*-ions and confirmed by manual interpretation of the MS/MS spectra (920.4535 (+1); matched by mass alone); Note, modifications to sequence identified by MS. Peptides identified by MS are underlined in the protein sequence. Note the non-tryptic *N*-terminal peptide (958.5200 (+3) *m/z*), suggesting the methionine at position 7 is the true *N*-terminus. Cysteine residue potentially involved in disulfide bond/homodimer formation is marked with (*).

We identified 2 proteins that were expressed from genes only encoded in the AES-1R genome (spots 26 and 43), and a further protein that was not contained within the PAO1 genome (spot 37). Previously hypothetical protein AES_7139 (spots 43 a-e; Figure 1) was the most abundant protein identified on the 2-DE gels of AES-1R and is present in multiple mass and p*I *variants. Variants exist at two masses, approximately 28 kDa and 16 kDa, with three p*I *variants at the higher mass (p*I *5.2, 5.6, and 6.0), and two p*I *variants at the lower mass (p*I *5.2 and 6.0). We subjected these spots to both MALDI-TOF MS peptide mass mapping and to LC-MS/MS for sequence characterization. We identified 9 peptide sequences that generated 90.8% sequence coverage for the predicted AES-1R gene (Figure 2). All variants generated near identical MALDI-MS spectra, suggesting the unusual migratory pattern on 2-DE gels are due to folding artifacts or poorly reduced disulfide bonds [31-33]. The AES_7139 translated gene sequence is predicted to encode a protein of 16.7 kDa and with a p*I *of 5.3, suggesting the higher mass variants may be homodimers or artifacts of the gel process. The sequence contains a single cysteine residue through which a disulfide could be formed, however under the reducing conditions used to conduct 2-DE, it is more likely that a gel artifact results in the spot pattern. One of the peptides sequenced by MS/MS displayed a non-tryptic *N*-terminus 8-GTYLFQYAQDKDYVLGVSDEQSGAK-32 (2782.4093 *m/z*) cleaved between Met-7 and Gly-8 that suggests either *N*-terminal processing, or that Met-7 is the true *N*-terminus. We subjected the AES_7139 protein sequence to BLAST search and showed that there is 100% amino acid sequence identity with a hypothetical protein (PA2G_05851) from *P. aeruginosa *PA2192 (Blastp score 311, query coverage 100%, e-value 2e-83), an isolate from a chronically infected CF patient in Boston. Other matches displayed similarity to ricin B-type lectins, suggesting the protein might be involved in carbohydrate binding. Importantly, however, no other *P. aeruginosa *genomes within the Swiss-Prot database contained AES_7139 homologs.

### Comparative gel-free proteomics of *P. aeruginosa *PAO1, PA14 and AES-1R using iTRAQ labelling and 2-DLC/MS-MS

Proteins from stationary phase cultures of *P. aeruginosa *AES-1R, PAO1 and PA14 were proteolytically digested, labeled using iTRAQ and analysed by 2-DLC-MS/MS. Multiple experiments were performed such that each strain was analysed in duplicate. Proteins with a ratio > 1.5 or < 0.67 (*p*-value < 0.05), and > 1.3 or < 0.77 (*p*-value < 0.01) were considered to be statistically differentially abundant. We identified a total of 1788 unique *P. aeruginosa *proteins, of which 1355 could be accurately quantified across the strains using iTRAQ. 162 proteins displayed significant differential abundance between the *P. aeruginosa *strains (Additional file 3). Of these, 60 were regulated identically between AES-1R compared to both PAO1 and PA14, 55 were only found in AES-1R versus PAO1, 39 were only found in AES-1R versus PA14 and 8 were differently abundant in AES-1R compared to both PAO1 and PA14, but in the opposite direction (e.g. more abundant in AES-1R compared to PAO1, but less abundant in AES-1R compared to PA14).

Functional analysis of the differently abundant proteins showed they could be clustered into 6 major groups: i) virulence determinants (including proteins involved in iron acquisition, phenazine biosynthesis and secreted factors); ii) membrane-associated proteins (including proteins involved in transport, antibiotic efflux, lipopolysaccharide (LPS) biosynthesis and outer membrane proteins [OMP]); iii) transcriptional and regulatory proteins; iv) proteins involved in translation; v) metabolic proteins; and vi) proteins of no known function. Of the 123 proteins found to be significantly altered in abundance between AES-1R and PAO1, 83 were present at elevated abundance in the AES-1R strain (40 present at reduced abundance); while of the 105 proteins significantly altered in abundance between AES-1R and PA14, 73 were present at increased abundance in AES-1R (32 present at reduced abundance). Within the functional clusters, proteins could also be classified by their relative abundance when compared between strains. For example, proteins involved in translation (predominantly ribosomal proteins) were overwhelmingly more abundant in AES-1R than either PA14 or PAO1 (Additional file 3).

We observed changes in abundance of several proteins thought to be involved in *P. aeruginosa *virulence. AES-1R displayed increased levels of chitinase ChiC, chitin-binding protein CbpD (PA0852), putative hemolysin (PA0122), hydrogen cyanide synthase HcnB (PA2194), while reduced abundance was detected for several other secreted proteins (e.g. Azurin, LasB elastase). It is important to note however, that these studies examined only intracellular proteins and do not reflect the amount of protein released into the extracellular environment during stationary phase growth. The LasB data do however, correlate with the phenotypic results observed from the elastase assays, where AES-1R produced more extracellular elastase function than PAO1, but less than PA14. Abundance differences could be detected for 4 proteins involved in the synthesis (PchEFG) or retrieval (FptA receptor) of the siderophore pyochelin. Interestingly, these were present at increased abundance in AES-1R when compared to PAO1, but reduced in AES-1R when compared to PA14. AES-1R also displayed reduced levels of other proteins involved in iron maintenance, including BfrA and BfrB bacterioferritin, although increased levels of a putative bacterioferritin (PA4880) were observed.

AES-1R displayed several changes associated with membrane transport and OMPs. Proteins with elevated abundance were associated with amino acid binding and small molecule transport (e.g. AotJ [PA0888], BraC [PA1074] and PhuT [PA4708]), as well as several lipoproteins, including OsmE (PA4876). AES-1R displayed highly elevated abundance of the type IV pilin structural subunit PilA (> 4-fold increase in abundance versus both PAO1 and PA14), as well as putative OMPs PA1689 and OmpA (PA3692), and the multi-drug efflux system protein MexX. The abundance difference for PilA in AES-1R may however be due to significant sequence differences between the 3 strains for this protein leading to an artificially inflated ratio (4.08 and 4.52 for PAO1 and PA14, respectively). Interestingly, a single AES-1R-specific protein (referred to here as AES_7145) with sequence similarity to an O-antigen/alginate biosynthesis protein UDP-*N*-acetyl-D-mannosaminuronate dehydrogenase, was also identified at very high levels in AES-1R. AES_7145 does not have a closely related homolog in either PAO1 or PA14 (< 50% sequence similarity to nearest match; data not shown) resulting in high iTRAQ reporter ratios (i.e. 3.835 versus PAO1 and 9.563 versus PA14). A sequence homolog was identified in the Liverpool CF epidemic strain LESB58 (PLES_19091 or WbpO; Blastp score 466, 97% sequence identity, e-value 9e-130). We also identified a second O-antigen biosynthesis protein, putative UDP-*N*-acetylglucosamine 2-epimerase (OrfK; PA14_23370), which appears to be unique to PA14. The presence of these proteins may reflect a difference in the LPS expressed in these strains. Other LPS proteins (e.g. lipid A 3-O-deacylase [PagL; PA4661]; 2-dehydro-3-deoxyphosphooctonate aldolase [KdsA; PA3636] and PslE [PA2235; unknown function in exopolysaccharide biosynthesis]) were also present in AES-1R at statistically significant altered abundance levels. PagL and KdsA however, were present at reduced abundance in AES-1R, along with several OMPs (OprD, OprG, OpmD, OprB2, OprQ and TolQ).

A number of proteins related to DNA replication, cell division and transcriptional regulation were observed to be differentially abundant between AES-1R and PAO1/PA14 (Additional file 3). The majority of these were present at increased abundance in AES-1R, including DNA-directed RNA polymerase alpha, beta and beta* (RpoABC; PA4238, PA4269 and PA4270), FtsH cell division protein (PA4751), Rho transcription termination factor (PA5239), histone-like protein HU (PA3940) and DNA gyrase subunit A (GyrA; PA3168). Inspection of the AES-1R GyrA protein sequence revealed an amino acid substitution of Thr83Ile (ACC- > ATC) (data not shown), which is a reported mutation in a number of CF clinical isolates showing quinolone resistance [34]. This mutation is also shared with the Liverpool epidemic strain LESB58 GyrA (PLES_19001). Interestingly, AES-1R showed increased abundance of the ferric uptake regulator (Fur; PA4764) in comparison to both PAO1 and PA14, although the degree of this increase was greater in comparison to PA14. Fur is the master regulator (repressor) of iron acquisition-related genes [35], and increased Fur levels are consistent with decreased abundances observed for several iron acquisition proteins (PchEFG, FptA, PA5217) when compared between AES-1R and PA14. Conversely however, we observed increased abundances of several of these proteins in AES-1R compared to PAO1, despite elevated Fur. Seven proteins were less abundant in AES-1R than in PAO1 or PA14, including 2 transcriptional regulators (MvaT [PA4315] and PA2667), and the RecG DNA helicase.

All differentially abundant proteins functionally clustered into the translation category were present at increased abundance in AES-1R. These were predominantly ribosomal proteins (13 proteins), although both elongation factors G and Ts were also present. Chaperonins GroEL, DnaK and HtpX were also present at elevated abundance in AES-1R. Forty-two proteins functionally classified as 'metabolic proteins' were present at altered abundance in AES-1R compared to PAO1 and PA14. Sub-clusters within this broad functional category were also readily identified. Ten proteins involved in fatty acid biosynthesis and metabolism were altered in abundance including 7 that were more abundant in AES-1R (FabB [PA1609], FabG [PA2967], acetyl-CoA carboxylase alpha [AccA; PA3639] and beta [AccD; PA3112], acyl carrier protein AcpP [PA2966], acyl-CoA thiolase [AspC; PA4785] and (R)-specific enoyl-CoA hydratase [PhaJ4; PA4015]). Twelve of the remaining proteins were functionally classified as playing a role in amino acid biosynthesis or degradation. Data for the three enzymes (ArcABC) of the arginine deiminase pathway showed all were present at elevated abundance in AES-1R compared to PA14, but were at lower levels in AES-1R than in PAO1. These results are in agreement with our 2-DE-based observations for AES-1R compared to PA14, where all three of ArcABC were present in higher abundance (or could only be observed) on gels derived from AES-1R. For AES-1R compared to PAO1 however, the data conflict to some degree since no difference between these two strains could be observed for arginine deiminase (ArcA), while carbamate kinase (ArcC) appeared to be significantly higher in AES-1R than PAO1. These results most likely reflect the ability to distinguish different mass and p*I *variants when using 2-DE-based approaches, whereas the iTRAQ peptide-based quantification technique reflects overall protein levels irrespective of chemical or physical protein post-translational modifications. This is further highlighted by our ability to identify 4 different forms of the ArcB ornithine carbamoyltransferase on 2-DE gels (Additional file 2).

The final functional group consisted of previously designated 'hypothetical' proteins, or proteins of no known function. Of these, one was encoded by a gene found only in AES_1R, while a second was only encoded by PA14. The AES-1R-specific hypothetical protein sequence (labelled here as AES_7165) was subjected to a BLAST sequence search and contained a region of sequence similarity to a type II restriction endonuclease (Cfr42I) from *Citrobacter freudii *(score 309, query coverage 100%, e-value 1e-82; data not shown). The other strain specific protein we identified was unique to PA14 (labelled PA14_53590). We were unable to find any sequence similarity between this hypothetical protein and any sequenced *Pseudomonas *or other bacterial gene/protein sequence.

### Comparison of gel-based and gel-free approaches for profiling *P. aeruginosa *strain differences

The overwhelming advantage of the gel-free approach was the ability to analyse the proteome at a much greater depth than a 2-DE gel-based approach. Gel-free analysis allowed the identification of 162 proteins that were altered in abundance between strains, while 2-DE enabled the identification of only 43 such proteins. Analysis of these 2 data sets showed that 22 proteins were identified as 'altered' by both 2-DE and iTRAQ 2-DLC/MS-MS (Additional file 2). The remaining 21 proteins identified by 2-DE were all characterized by gel-free means, and the majority showed the same *n*-fold change, but could not be included since they did not reach the required rigorous statistical cut-off for significance. The data do however; show a typical distribution for comparison of 2-DE and 2-DLC/MS-MS, where the majority of both identifications and quantified changes can be observed using gel-free means, yet some unique data (typically relating to protein degradation/fragmentation; e.g. OmpA or other modifications) are obtained using gel-based approaches. For the overlapping 22 proteins, we compared the data derived from both 2-DE densitometry and iTRAQ gel-free techniques (Additional file 4; 44 comparisons--AES-1R compared to PAO1 and PA14). The increases and decreases in abundance were generally consistent across techniques for most proteins (22/44 comparisons and a further 11/44 where the iTRAQ ratios were not statistically significant for inclusion). 9/44 were detected as changing by iTRAQ 2-DLC/MS-MS but were apparently not changing on 2-DE gels, while only 2/44 comparisons showed opposite changes. These last two groups contained many proteins that appear as multiple protein 'spots' on 2-DE gels (e.g. ArcB) and thus it is difficult to gauge the overall abundance of those proteins by the gel-based approach.

## Discussion

This study compared proteome profiles of 3 *P. aeruginosa *strains to identify candidate proteins that may be specific to the acute, transmissible CF strain AES-1R. Proteins identified in the AES-1R isolate may reflect adaptations specific to the environmental niche of this organism and that could provide a colonization advantage in the CF lung. A number of virulence determinants differed in abundance between strains, including secreted factors, siderophore and pigment producing enzymes, and oxidative stress response proteins. *P. aeruginosa *is known to produce a number of secreted virulence factors including toxins, proteases and binding proteins, many of which are under QS regulation [36,37]. AES-1R protein profiles displayed elevated abundances of chitinase ChiC, chitin-binding protein CbpD, and putative hemolysin (PA0122), while the major secreted protease elastase LasB was increased in virulent PA14. Microarray studies have shown increased expression of *hcnB *and *cbpD *in mucoid *P. aeruginosa *compared to non-mucoid [38]. Since AES-1R is non-mucoid, it is possible that these proteins are more abundant in CF isolates irrespective of mucoidy when compared to non-CF strains. Comparisons of a chronic, rather than acute, CF isolate with PAO1 also showed increased *cbpD *gene expression [25]. Putative hemolysin (PA0122) is highly expressed in non-mucoid CF clinical isolates and binds oxidised low-density lipoprotein from human serum present in the CF lung [39,40]. Increased chitinase production may provide AES-1R with an enhanced ability to degrade lung connective tissues [41]. We also observed elevated abundance in AES-1R of PasP (PA0423), a small protease that cleaves collagens and a virulence determinant in *P. aeruginosa*-associated corneal infections [42]. PasP has been described as an immunogen in 4 CF patient sera [43]. Importantly, it must be noted that our data reflect intracellular levels of these predominantly secreted proteins, suggesting either altered expression or impaired secretion. We were able to detect higher elastase and total protease activities for AES-1R compared to PAO1, while total hemolysin activity was approximately the same. Similar to the proteomics data, we observed reduced elastase activity for AES-1R compared to PA14.

*P. aeruginosa *produces compounds such as pyocyanin (induction of oxidative damage and pro-inflammatory effect reviewed by [44]), hydrogen cyanide (decoupling host cell respiration), and siderophores for the scavenging of iron. Pyocyanin exerts multiple detrimental effects on the host, primarily through its ability to produce reactive oxygen species, and is capable of repressing transcription of host oxidative stress defense proteins [45], interfering with metabolism [46], inhibiting beating of cilia [47], proinflammatory action [48], neutrophil apoptosis [49] and increased levels correlate with CF pulmonary exacerbations [50]. *P. aeruginosa *possesses two operons (*phzA1B1C1D1E1F1G1 *and *phzA2B2C2D2E2F2G2*) for the synthesis of phenazine-carboxylic acid (PCA), which is then further processed by PhzM to 1-hydroxyphenazine (1-HP) and finally, PhzS to pyocyanin. These intermediates also exhibit cytotoxic effects on the host [47,51,52]. We observed elevated levels of PhzS in AES-1R compared to PAO1 (gel-free approach) and PA14 (2-DE gel-based analysis), yet a decrease in comparative PhzB2 levels. Increased PhzS may reflect elevated 1-HP to pyocyanin, which is supported by several studies showing pyocyanin production is enhanced in CF strains [53,54] and reflected in AES-1R phenotypic data compared to PAO1 (Table 1). Decreased PhzB2 abundance may reflect differential induction of the 2 Phz operons across strains [47,51,52,55].

Iron acquisition via siderophore production is critical for successful colonization of the CF lung and for providing *P. aeruginosa *with a distinct competitive advantage over other pathogens. The host generally limits free iron by sequestration via transferrin, ferritin and lactoferrin. The CF lung may contain higher iron availability (CF, 13-32 μmol.L^-1 ^c.f. normal 0-13.2 μmol.L^-1 ^[56]), most likely due to tissue damage resulting from an exaggerated inflammatory response. *P. aeruginosa *produces the pyochelin and pyoverdine siderophores to acquire iron from the environment and the later is thought to be a major contributor in the CF lung [57]. We observed increases in abundance of pyochelin synthetases (PchEF) in AES-1R compared to PAO1. Transcriptomic studies have also shown increased expression of *pchEF *in a chronic CF isolate [25]. In contrast, PA14 produced even greater levels of PchEF, as well as pyochelin synthetase PchG and the Fe(III)-pyochelin outer membrane receptor FptA. This confirms that iron acquisition is important in general virulence as well as in the specific CF lung micro-environment. Other proteins involved in iron uptake and storage were differentially abundant between the strains studied. The iron storage bacterioferritins BfrA and BfrB were decreased in abundance in AES-1R, however a putative bacterioferritin PA4880 was markedly increased in abundance suggesting it may be the preferred storage protein in this isolate. These data correlated with previous transcript analyses--increased expression of *pa4880 *was observed in a chronic CF isolate compared to PAO1 [25], while decreased *bfrB *was observed under iron starvation suggesting AES-1R is adapted to an iron-limited environment [58].

CF-associated AES-1R was the only strain with detectable flagellin in the protein extracts analysed by 2-DE. AES-1R FliC has significant protein sequence differences compared with PAO1 and PA14, and has greater sequence similarity with the type A flagellin of strain PAK (Additional file 5). Increased flagellin in AES-1R is consistent with our phenotypic data for swimming motility and with previous work showing AES-1 isolates displayed greater motility than non-clonal CF isolates [59]. Several differences in the OMP profile of AES-1R were observed. The loss of OprD in AES-1R is characteristic of carbapenem antibiotic resistance [60]. Decreased OprG expression was originally associated with increased fluoroquinolone resistance [61], however a recent study showed no significant difference in the antibiotic susceptibility profile of an *oprG*-deficient strain [62]. *ΔoprG P. aeruginosa *do show a 3-fold decrease in cytotoxicity toward the human bronchial epithelial cell line HBE, however transcriptomics revealed a rapid down-regulation of *oprG *in wild-type *P. aeruginosa *upon interaction with these cells [62]. MexX, a component of the MexXY-OprM multidrug efflux transporter, was markedly increased in abundance in AES-1R and is known to confer resistance to a number of antibiotics including erythromycin, fluoroquinolones, aminoglycosides and the ß-lactams, cefepime and ceftobiprole [63-66], correlating well with the antibiotic resistance associated with CF infections. Quinolones are the antibiotic of choice for treatment of *P. aeruginosa *CF lung infections and resistance to this class of drug can result from mutations within DNA gyrase GyrA (PA3168), which is essential for DNA replication. The AES-1R *gyrA *gene sequence revealed an amino acid substitution (Thr83Ile) previously reported to result in quinolone resistance [34] and observed in the Liverpool epidemic strain LESB58. Increased abundance of PA5178 (putative LysM domain protein), a protein containing a domain with predicted bacterial wall degradation properties may suggest a potential advantage against competing pathogens. *P. aeruginosa *is predicted to contain approximately 185 genes encoding lipoproteins [67]. A number of lipoproteins were observed at increased abundance in AES-1R. Induction of lipoprotein genes has been associated with an excessive proinflammatory response in lung epithelial cells via Toll-like receptor 2 [68]. OprI (PA2853) is an immunogenic lipoprotein that has been proposed as part of a multivalent vaccine [69]. We observed reduced OprI abundance in AES-1R, which may influence the efficacy of an OprI-based vaccine.

LPS is a major virulence factor that is involved in initiating the pro-inflammatory response in the host. *P. aeruginosa *strains produce different LPS types, which are currently classified into 20 serotypes. Strain-specific enzymes involved in O-antigen synthesis were identified in AES-1R (UDP-*N*-acetyl-D-mannosaminuronate dehydrogenase (AES_7145)) and PA14 (putative UDP-*N*-acetylglucosamine 2-epimerase OrfK (PA14_23370)) and these may reflect serotype differences. LPS has also been implicated in evasion of the host immune response and antibiotic resistance in CF lung infection [70,71]. The LPS modification enzyme lipid A 3-O-deacylase PagL (PA4661) catalyses the production of a penta-acylated lipid A [72]. Reduced abundance of PagL in AES-1R (compared with PA14) is consistent with previous findings showing a third of *P. aeruginosa *isolates from CF patients with severe lung disease produced hexa- or hepta-acylated lipid A, due to a decrease in 3-O-deacylase activity [71]. A consistent finding in AES-1R was increased abundance of enzymes involved in fatty acid biosynthesis. Further weight is given to this evidence from transcriptomic results showing increased expression levels of fatty acid biosynthesis enzymes in a chronic CF isolate compared to PAO1 [25]. This collection of pathways supplies an essential building block used in a number of cell processes, particularly membrane synthesis and provides the acyl groups necessary for the synthesis of acyl-homoserine lactones (AHLs) [73], the autoinducer signal molecules necessary for QS.

Our studies allowed the identification of previously hypothetical proteins, particularly those unique to AES-1R. A protein of unknown function (AES_7139) was the most abundant observed on the 2-DE profiles of AES-1R. AES_7139 is found in a large region of the AES-1R genome (AES_6966 to _7152) containing nearly entirely AES-1R-specific coding sequences [30]. This protein sequence could only be found by BLAST search in a second CF-associated *P. aeruginosa *isolate (hypothetical protein PA2G_05851 from *P. aeruginosa *PA2192; [19]), and contains a ricin-type lectin conserved domain that is associated with carbohydrate binding. Analysis of mucin glycosylation in the sputum of CF patients has shown altered glycan patterns, consisting of increased sialylation and reduced sulfation and fucosylation [74,75]. Since mucin glycan structures may be altered, specific proteins such as AES_7139/PA2G_05851 could be necessary for binding lung epithelium. Certainly the overall abundance detected here suggests a central role for this protein in the environmental survival of AES-1R and a potential role in early infection. A further two AES-1R-specific hypothetical proteins (AES_7104 and AES_7165) were also identified.

Approximately a third of the theoretical *P. aeruginosa *proteome (1788 proteins) was identified by gel-free 2-DLC/MS-MS, with 75% of these providing sufficient data for accurate quantitation. The 2-DE approach however does allow for the relative abundance of individual proteins to be compared within a sample (for example, AES_7139 as the most abundant 'spot' in comparison to all other protein spots). 2-DE also provides additional information regarding protein variants, processing or post-translational modification, as well as facilitating the identification of proteins with sequence differences compared to genomically characterized relatives or highlighting mis-annotations within a gene sequence (e.g. flagellin (FliC/FlaA) and type IV pilin types (PilA)). Transcriptomic analyses are able to quantitate gene expression accurately up to 4.7 orders of magnitude [76], however proteomic strategies such as iTRAQ only achieve measurement of around 2 orders of magnitude. Technical limitations of the iTRAQ method may lead to an underestimation of the magnitude of change [77], while many proteins are below the limit of detection by 2-DE. Clear examples of iTRAQ ratio underestimation are seen in proteins that are unique to a particular strain, such as AES_7165 (unique to AES-1R), which despite being absent in PAO1 and PA14 only achieved measured ratios of 4.15 and 4.90, respectively.

## Conclusions

A complementary proteomic approach combining gel-based (2-DE) and gel-free (2-DLC-MS/MS with iTRAQ tags) techniques was employed to quantitatively compare the proteomes of *P. aeruginosa *strains PAO1, PA14 and AES-1R (an acute, transmissible CF isolate). Proteins associated with AES-1R belonged to a variety of functional groups including virulence factors, antibiotic resistance, LPS and fatty acid biosynthesis, and several hypothetical proteins. Proteins involved in the acquisition of iron were elevated in AES-1R compared to PAO1, while being decreased compared to PA14. These results confirm that CF-associated *P. aeruginosa *strains express a unique protein profile indicative of phenotypic adaptations to their environment and that provide traits conferring an advantage in colonization of the CF lung micro-environment. Identification of the proteins used by transmissible strains will aid in the elucidation of novel intervention strategies to reduce the burden of *P. aeruginosa *infection in CF patients.

## Authors' contributions

NH aided in experimental design and carried out the protein analyses, including 2-DE, 2-DLC-MS/MS and data analysis, and drafted the manuscript. NS and NBM undertook LC-MS and peptide mass mapping experiments and data analysis. NH and CHa performed phenotypic analyses. BR, CH, and JM contributed to the coordination of the study and data interpretation. BC provided MS instrument-specific training and guidance on experimental design. SJC conceived the study, aided in the experimental design and, coordination, undertook data analysis and interpretation, and drafted the manuscript. All authors approved the final manuscript.

## Supplementary Material

Additional file 1 **Growth curves for *P. aeruginosa *AES-1R, PAO1 and PA14 grown to stationary phase in LB medium**. Dotted line and *, harvest time for PAO1 and PA14; #, for AES-1R. Click here for file

Additional file 2 **Table containing identification of differentially abundant proteins in *P. aeruginosa *AES-1R compared to PAO1 and PA14 using 2-DE**. Click here for file

Additional file 3 **Table containing identification of differentially abundant proteins in *P. aeruginosa *AES-1R compared to PAO1 and PA14 using iTRAQ**. Click here for file

Additional file 4 **Figure showing overlap of identified and quantified proteins by 2-DE and 2-DLC/MS with iTRAQ**. Table showing relative abundance changes for 22 proteins quantified by both 2-DE and iTRAQ. Click here for file

Additional file 5 **Protein sequence alignment of Flagellin (FliC/FlaA) of *P. aeruginosa *strains used in this study (AES_1954, PA1092, and PA14_50290) and including an additional sequence from strain PAK with a known type A flagellin**. The flagellin sequence of strain AES-1R has higher sequence similarity with the shorter A type flagellin of strain PAK (95%), while the type B flagellins of strains PA14 and PAO1 are almost identical with only a single amino acid difference. Click here for file
