# Supplementary figures and images for: Proteomic profiling of Pseudomonas aeruginosa AES-1R, PAO1 and PA14 reveals potential virulence determinants associated with a transmissible cystic fibrosis-associated strain
Source: BMC Microbiol. 2012 Jan 22;12:16. doi: 10.1186/1471-2180-12-16 (PMC3398322; doi:10.1186/1471-2180-12-16)

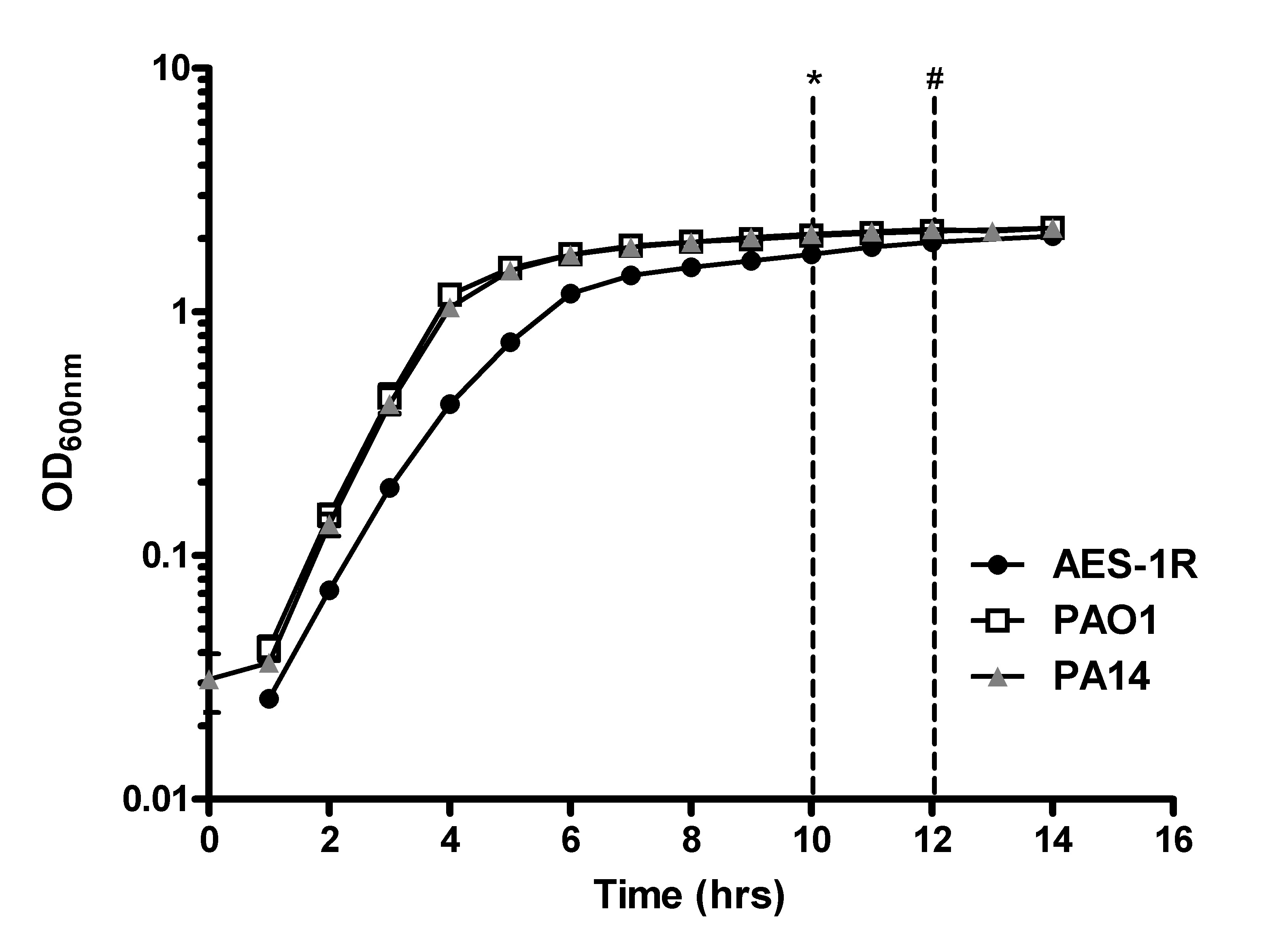

Supplement: Additional file 1 — Growth curves for P. aeruginosa AES-1R, PAO1 and PA14 grown to stationary phase in LB medium. Dotted line and *, harvest time for PAO1 and PA14; #, for AES-1R. [file 1471-2180-12-16-S1.JPEG]
