# Supplementary material for: Proteomic profiling of Pseudomonas aeruginosa AES-1R, PAO1 and PA14 reveals potential virulence determinants associated with a transmissible cystic fibrosis-associated strain
Source: BMC Microbiol. 2012 Jan 22;12:16. doi: 10.1186/1471-2180-12-16 (PMC3398322; doi:10.1186/1471-2180-12-16)
Supplement: Additional file 2 — Table containing identification of differentially abundant proteins in P. aeruginosa AES-1R compared to PAO1 and PA14 using 2-DE. [file 1471-2180-12-16-S2.PDF]

Additional File 2. Identification of differentially abundant proteins in *P. aeruginosa* AES-1R compared to PAO1 and PA14 using 2-DE.

Spot No.	AES No.	PAO1 No.	PA14 No.	Protein Name	Gene Name	No. Pept.	Seq. Cov. (%)	MASC	p*I*	Mass	AES-1R v PA14	p-value	AES-1R v PAO1	p-value	AES-1R spot density (mean)	SEM	PA14 spot density (mean)	SEM	PAO1 spot density (mean)	SEM
1	AES_5124	PA3648	PA14_17150	Putative Outer Membrane Antigen Opr86	*opr86*	15	27	136	5.08	86737	+	+	1.54	0.011	38894	2906	n.d.	n.d.	25226	1509
2	AES_5685	PA4221	PA14_09340	Fe(III)-Pyochelin Outer Membrane Receptor FptA	*fptA*	17	29	196	5.86	79943	0.66	0.037	0.82	0.245	6932	842	10529	1034	8448	819
3	AES_2690	PA1596	PA14_43850	Heat Shock Protein HtpG	*htpG*	18	34	214	5.10	72125	2.45	0.000	1.40	0.033	51610	2520	21094	3163	36978	4273
4	AES_4284	PA4761	PA14_62970	Heat Shock Protein DnaK	*dnaK*	20	42	267	4.79	68361	+	+	2.52	0.003	27874	2191	n.d.	n.d.	11065	849
5	AES_4660	PA3162	PA14_23330	30S Ribosomal Protein S1	*rpsA*	10	27	108	4.83	62077	+	+	1.52	0.002	60298	3025	n.d.	n.d.	39738	1971
6	AES_6039	PA4595	PA14_60800	Putative ABC Transporter, ATP-Binding Protein		17	36	228	5.47	61283	1.73	0.000	0.99	0.863	22639	771	13064	424	22940	1455
7	AES_6943	PA5556	PA14_73260	F_0F_1 ATP Synthase Subunit Alpha	*atpA*	15	32	123	5.33	55359	2.36	0.000	1.43	0.008	79769	1758	33780	3009	55826	4418
8	AES_6394	PA4974	PA14_65750	Putative Outer Membrane Efflux Protein OmpH	*ompH*	25	62	305	5.80	53373	0.67	0.029	0.88	0.270	17434	1071	26211	2436	19752	1552
9a	AES_3607	PA2300	PA14_34870	Chitinase ChiC	*chiC*	12	39	142	5.22	53009	1.88	0.001	1.42	0.001	11039	440	5874	648	7760	342
9b	AES_3607	PA2300	PA14_34870	Chitinase ChiC	*chiC*	10	39	145	5.22	53009	2.76	0.038	1.18	0.215	110011	7255	39891	128	93199	11973
9c	AES_3607	PA2300	PA14_34870	Chitinase ChiC	*chiC*	13	34	116	5.22	53009	1.51	0.021	0.85	0.316	34031	2788	22508	907	40121	4688
10a	AES_5843	PA4385	PA14_57010	GroEL protein	*groEL*	17	54	245	4.91	55403	+	+	2.37	0.002	166092	9867	n.d.	n.d.	69959	1224
10b	AES_5843	PA4385	PA14_57010	GroEL protein	*groEL*	10	20	64	4.91	55403	0.87	0.298	0.67	0.065	21729	1842	24948	1079	32194	2065
11	AES_3596	PA2291	PA14_34960	Carbohydrate-Binding Porin	*oprB2*	10	22	107	5.36	50546	1.00	0.984	0.73	0.046	16114	573	16170	2548	22185	1925
12	AES_0595	PA0291	PA14_03800	Anaerobically-Induced Outer Membrane Porin OprE	*oprE*	14	39	162	8.67	49637	0.80	0.010	0.73	0.004	64515	3187	80504	2890	88985	4232
13	AES_6913	PA5554	PA14_73240	F_0F_1 ATP Synthase Subunit Beta	*atpD*	20	65	197	4.98	49469	1.75	0.003	1.24	0.072	133651	4992	76230	8817	107719	9865
14	AES_2965	PA1800	PA14_41250	Trigger Factor	*tig*	26	68	279	4.83	48552	+	+	1.75	0.000	29472	766	n.d.	n.d.	16835	489
15	AES_1776	PA0958	PA14_51880	Basic Amino Acid, Basic Peptide and Imipenem Outer Membrane Porin OprD Precursor	*oprD*	16	57	187	4.96	48331	-	-	-	-	n.d.	n.d.	341793	20021	247569	18069
16	AES_4165	PA2760	PA14_28400	Putative Outer Membrane Porin OprD Family	*oprQ*	15	43	162	5.54	46822	1.50	0.038	1.06	0.637	232048	22070	154198	2112	218043	17446
17	AES_1670	PA5171	PA14_68330	Arginine Deiminase	*arcA*	10	36	87	5.43	46422	3.05	0.047	1.06	0.284	21965	1333	7193	304	20662	117
18	AES_0447	PA4217	PA14_09400	Flavin-Containing Monooxygenase	*phzS*	9	28	97	5.60	43504	+	+	0.81	0.304	17949	1517	n.d.	n.d.	27567	738

19	AES_1954	PA1092	PA14_50290	Flagellin Type B	*fliC*	13	50	139	4.81	39976	+	+	+	+	242016	23027	n.d.	n.d.	n.d.	n.d.
20a	AES_1669	PA5172	PA14_68340	Ornithine Carbamoyltransferase	*arcB*	10	29	112	6.13	38084	0.93	0.377	1.86	0.016	11759	700	12633	972	6327	29
20b	AES_1669	PA5172	PA14_68340	Ornithine Carbamoyltransferase	*arcB*	14	33	101	6.13	38084	1.05	0.799	+	+	20053	225	19068	7977	n.d.	n.d.
20c	AES_1669	PA5172	PA14_68340	Ornithine Carbamoyltransferase	*arcB*	11	33	101	6.13	38084	1.15	0.503	+	+	19079	88	16543	2558	n.d.	n.d.
20d	AES_1669	PA5172	PA14_68340	Ornithine Carbamoyltransferase	*arcB*	15	39	110	6.13	38084	0.68	0.262	+	+	10986	1041	16039	2504	n.d.	n.d.
21a	AES_2937	PA1777	PA14_41570	Major Outer Membrane Porin OprF Precursor	*oprF*	13	36	124	4.98	37616	0.86	0.077	1.09	0.170	216522	6893	252913	13691	198929	7040
21b	AES_2937	PA1777	PA14_41570	Major Outer Membrane Porin OprF Precursor	*oprF*	14	45	154	4.98	37616	2.81	0.002	1.69	0.031	447845	36289	159236	15124	264747	51724
21c	AES_2937	PA1777	PA14_41570	Major Outer Membrane Porin OprF Precursor	*oprF*	10	39	75	4.98	37616	1.19	0.323	1.56	0.062	398774	49786	334932	29950	254872	6635
22	AES_6664	PA5243	PA14_69240	Delta-Aminolevulinic Acid Dehydratase	*hemB*	8	31	94	5.06	37014	3.22	0.015	1.28	0.321	23009	3275	7155	746	17961	3324
23	AES_6636	PA5217	PA14_68900	Putative Iron ABC Transporter, Periplasmic Iron-Binding Protein		13	40	151	6.02	36270	+	+	+	+	13850	549	n.d.	n.d.	n.d.	n.d.
24	AES_1668	PA5173	PA14_68350	Carbamate Kinase	*arcC*	13	40	131	5.25	33059	+	+	1.33	0.041	27345	801	n.d.	n.d.	20593	2091
25	AES_2395	PA4352	PA14_56590	Putative Universal Stress Protein		18	75	208	5.92	30953	1.58	0.001	1.22	0.175	26738	1144	16886	782	21923	2698
26	AES_7104	-	-	Hypothetical Protein		7	30	58	6.60	29999	+	+	+	+	8855	182	n.d.	n.d.	n.d.	n.d.
27	AES_5171	PA3692	PA14_16630	Putative Outer Membrane Protein (fragment)	*ompA*	8	45	106	9.45	28497	1.43	0.028	1.04	0.784	71131	2188	49747	5816	68627	8171
28	AES_4630	PA4067	PA14_11270	Outer Membrane Protein OprG Precursor	*oprG*	9	46	90	4.85	25178	0.48	0.000	0.56	0.004	111002	4555	233567	7945	199974	13374
29	AES_5942	PA4495	PA14_58330	Conserved Hypothetical Protein		12	45	157	5.79	24864	+	+	+	+	42315	4756	n.d.	n.d.	n.d.	n.d.
30	AES_2602	PA2575	PA14_30800	Hypothetical Protein		7	42	111	5.96	22208	1.08	0.403	0.75	0.019	17584	1331	16282	249	23482	296
31	AES_4871	PA3326	PA14_21030	Putative ATP-Dependent Clp Protease Proteolytic Subunit		8	38	89	5.44	22128	1.36	0.002	0.60	0.000	38126	1486	27968	999	51417	7184
32	AES_1147	PA3529	PA14_18690	Putative Alkyl Hydroperoxide Reductase Subunit		9	48	119	5.37	21808	+	+	1.30	0.010	30248	1482	n.d.	n.d.	23240	1095
33	AES_0809	PA0423	PA14_05510	Periplasmic Protein PasP	*pasP*	11	70	120	6.09	20764	+	+	1.60	0.000	54319	1889	n.d.	n.d.	33988	1032
34	AES_0223	PA0139	PA14_01710	Alkyl Hydroperoxide Reductase Subunit C	*ahpC*	8	41	96	5.89	20529	+	+	0.91	0.074	27891	1032	n.d.	n.d.	30647	192
35	AES_6067	PA4880	PA14_64520	Putative Bacterioferritin		8	41	108	4.64	20205	+	+	1.15	0.358	13399	814	n.d.	n.d.	11605	1255
36	AES_0365	PA2331	PA14_34460	Putative Alkylhydroperoxidase		7	32	91	5.06	19322	+	+	0.82	0.315	25262	17	n.d.	n.d.	30758	2963
37	AES_0626	-	PA14_04010	Hypothetical Protein		6	37	78	5.09	19221	+	+	+	+	40083	928	n.d.	n.d.	n.d.	n.d.
38	AES_6094	PA4661	PA14_61650	Lipid A 3-O-Deacylase	*pagL*	6	31	93	5.87	18382	-	-	-	-	n.d.	n.d.	30085	1103	n.d.	n.d.
39	AES_4848	PA3309	PA14_21220	Hypothetical Protein	*uspK*	10	68	169	5.50	16486	1.81	0.000	0.85	0.034	73585	308	40673	253	86279	993
40	AES_1663	PA5178	PA14_68400	Putative LysM Domain Protein		9	62	120	5.46	14785	+	+	1.47	0.004	104301	4826	n.d.	n.d.	71004	2514

41	AES_6717	PA5288	PA14_69810	Nitrogen Regulatory Protein PII	*glnK*	8	62	151	5.41	12279	+	+	0.98	0.878	33953	3345	n.d.	n.d.	34514	199
42	AES_5710	PA4236	PA14_09150	Catalase KatA	*katA*	13	40	158	6.14	55496	0.53	0.038	0.46	0.029	7514	607	14072	1940	16169	4875
43a	AES_7139	-	-	Hypothetical Protein		5	44	1553	5.28	16797	+	+	+	+	396516	40142	n.d.	n.d.	n.d.	n.d.
43b	AES_7139	-	-	Hypothetical Protein		4	38	390	5.28	16797	+	+	+	+	51651	4171	n.d.	n.d.	n.d.	n.d.
43c	AES_7139	-	-	Hypothetical Protein		5	44	2055	5.28	16797	+	+	+	+	402187	15670	n.d.	n.d.	n.d.	n.d.
43d	AES_7139	-	-	Hypothetical Protein		6	78	1527	5.28	16797	+	+	+	+	421865	21623	n.d.	n.d.	n.d.	n.d.
43e	AES_7139	-	-	Hypothetical Protein		3	20	563	5.28	16797	+	+	+	+	375193	22555	n.d.	n.d.	n.d.	n.d.

Proteins were identified by peptide mass mapping and quantitated by densitometry of 2-DE gels. Spot No. refers to spot number from 2-DE gels (Figure 1). AES No., PAO1 No. and PA14 No. refer to translated ORF number from AES-1, PAO1 and PA14 genome sequence. No. Pept. refers to number of matching peptide masses from MALDI-TOF MS analysis of trypsin digests; Seq. Cov. (%), % of the sequence covered by matching peptides; MASC., MASCOT score derived from those matching peptides. p*I* and mass predicted from translated ORFs. AES-1R v PA14 (PAO1), *n*-fold difference in abundance in AES-1R compared to PA14 and PAO1. Proteins elevated in abundance in AES-1R display an *n*-fold change >1 (shaded red if p-value < 0.05), while proteins reduced in abundance in AES-1R display an *n*-fold change <1 (shaded blue). Spot density (mean), mean spot densitites in ppm as detected from 2-DE gel image analysis using PD-Quest software from 6 replicate 2-DE gels. SEM, standard error of the mean. +, protein only detected in AES-1R versus either PA14 or PAO1; -, protein not detected in AES-1R versus either PA14 or PAO1. n.d., not detected.
