# Supplementary material for: Proteomic profiling of Pseudomonas aeruginosa AES-1R, PAO1 and PA14 reveals potential virulence determinants associated with a transmissible cystic fibrosis-associated strain
Source: BMC Microbiol. 2012 Jan 22;12:16. doi: 10.1186/1471-2180-12-16 (PMC3398322; doi:10.1186/1471-2180-12-16)
Supplement: Additional file 3 — Table containing identification of differentially abundant proteins in P. aeruginosa AES-1R compared to PAO1 and PA14 using iTRAQ. [file 1471-2180-12-16-S3.PDF]

Additional File 3. Identification of differentially abundant proteins in *P. aeruginosa* AES-1R compared to PAO1 and PA14 using iTRAQ

AES-1R No.	PAO1 No.	PA14 No.	Protein Name	Gene Name	AES-1R v PAO1	p-value	No. Pept.	Seq. Cov. %	AES-1R v PA14	p-value	No. Pept.	Seq. Cov. %
**Virulence Factors (Iron Acquisition / Secreted Factors / Oxidative Stress)**												
AES_5583	PA4078	PA14_11140	Putative Nonribosomal Peptide Synthetase		1.9025	0.0001	87	38.95				
AES_5685	PA4221	PA14_09340	Fe(III)-Pyochelin Outer Membrane Receptor FptA	*fptA*					0.5701	0.0003	2	1.67
AES_5691	PA4224	PA14_09290	Pyochelin Biosynthetic Protein PchG	*pchG*					0.4965	0.0002	8	3.72
AES_3620	PA4225	PA14_09280	Pyochelin Synthetase PchF	*pchF*	1.9023	0.0058	12	11.28	0.6873	0.0123	21	12.00
AES_5384	PA4226	PA14_09270	Pyochelin Synthetase PchE	*pchE*	1.4059	0.0001	32	17.94	0.6589	0.0000	32	17.94
AES_1145	PA3531	PA14_18670	Bacterioferritin	*bfrB*	0.4197	0.0084	9	28.48	0.7602	0.0314	6	29.75
AES_5706	PA4235	PA14_09160	Bacterioferritin	*bfrA*	0.5509	0.0015	30	51.30	0.8367	0.0383	18	51.30
AES_6067	PA4880	PA14_64520	Putative Bacterioferritin		3.4003	0.0034	6	44.07				
AES_0447	PA4217	PA14_09400	Flavin-Containing Monooxygenase PhzS	*phzS*	1.4789	0.0059	17	41.54				
AES_3118	PA1900	PA14_39960	Probable Phenazine Biosynthesis Protein PhzB2	*phzB2*	0.5762	0.0198	16	35.80	0.3301	0.0041	13	50.00
AES_0187	PA0122	PA14_01490	Putative Hemolysin		1.6038	0.0079	14	72.79	3.0546	0.0049	43	20.59
AES_1561	PA0852	PA14_53250	Chitin-Binding Protein CbpD Precursor	*cbpD*	1.9528	0.0295	35	52.44	2.7966	0.0000	35	52.44
AES_3607	PA2300	PA14_34870	Chitinase ChiC	*chiC*	1.5201	0.0040	54	63.98				
AES_3490	PA2194	PA14_36320	Hydrogen Cyanide Synthase	*hcnB*	1.4619	0.0013	5	4.74	1.6024	0.0364	5	4.74
AES_5019	PA3479	PA14_19100	Rhamnosyltransferase Chain A	*rhlA*	0.6152	0.0062	4	15.59				
AES_6349	PA4922	PA14_65000	Azurin Precursor	*azu*	0.2733	0.0000	21	58.78				
AES_5204	PA3724	PA14_16250	Elastase LasB	*lasB*					0.5326	0.0059	3	10.64
AES_0365	PA2331	PA14_34460	Putative Alkylhydroperoxidase						2.0023	0.0004	9	22.58
AES_5710	PA4236	PA14_09150	Catalase KatA	*katA*	0.3196	0.0037	69	49.38				
AES_0796	PA0413	PA14_05390	Still Frameshift Probable Component of Chemotactic Signal Transduction System	*chpA*	0.7630	0.0093	17	8.13				

Membrane-Associated Proteins (Periplasmic Transport / Lipoproteins / LPS Biosynthesis and Modification / Outer Membrane Proteins)

AES_6636	PA5217	PA14_68900	Putative Iron ABC Transporter, Periplasmic Iron-Binding Protein		1.7872	0.0013	14	21.39	1.6260	0.0110	14	22.89
AES_1650	PA0888	PA14_52790	Arginine / Ornithine Binding Protein AotJ	*aotJ*	1.8907	0.0059	21	53.28	1.3223	0.0153	21	53.28
AES_1929	PA1074	PA14_50520	Branched-Chain Amino Acid Transport Protein	*braC*	1.6621	0.0127	23	53.62	1.4079	0.0053	23	53.62
AES_6160	PA4708	PA14_62300	Putative Periplasmic Binding Protein	*phuT*	2.3668	0.0048	2	10.44				
AES_2411	PA1342	PA14_46910	Putative Binding Protein Component of ABC Transporter						1.3836	0.0015	43	61.26
AES_0417	PA0300	PA14_03920	Polyamine Transport Protein	*spuD*	0.6616	0.0286	18	29.43	0.6357	0.0338	10	11.99
AES_4695	PA3190	PA14_22980	Putative Binding Protein Component of ABC Sugar Transporter		0.6061	0.0040	13	26.90				
AES_1831	PA1011	PA14_51260	Putative Lipoprotein		2.1085	0.0012	20	51.01	1.4617	0.0149	20	51.01
AES_0326	PA4639	PA14_61390	Putative Lipoprotein		1.8852	0.0047	8	26.15	1.2424	0.0183	8	26.15
AES_3554	PA4876	PA14_64480	Osmotically Inducible Lipoprotein OsmE	*osmE*	2.1174	0.0068	14	52.63				
AES_5170	PA3691	PA14_16640	Putative Lipoprotein						1.5904	0.0318	10	34.33
AES_4304	PA2853	-	Outer Membrane Lipoprotein OprI Precursor	*oprI*					0.4879	0.0000	137	39.76
AES_7145	-	-	UDP-N-Acetyl-D-Mannosaminuronate Dehydrogenase		3.8350	0.0080	4	21.30	9.5627	0.0089	4	21.30
-	-	PA14_23370	Putative UDP-N-Acetylglucosamine 2-Epimerase	*orfK*					0.3550	0.0000	10	32.28
AES_6094	PA4661	PA14_61650	Lipid A 3-O-Deacylase	*pagL*					0.5982	0.0000	5	17.92
AES_4598	-	PA14_58730	Type IV Pilin Structural Subunit	*pilA*	4.0834	0.0032	16	43.67	4.5151	0.0021	16	43.67
AES_2827	PA1689	PA14_42670	Putative Membrane Protein		3.4860	0.0481	6	12.00	1.7944	0.0008	10	9.00
AES_3290	PA2019	PA14_38395	Resistance-Nodulation-Cell Division (RND) Multidrug Efflux Membrane Fusion Protein Precursor	*mexX*	2.8937	0.0022	5	28.03	2.1277	0.0132	5	28.03
AES_5171	PA3692	PA14_16630	Putative Outer Membrane Protein OmpA		3.9761	0.0006	44	49.43	2.3183	0.0000	40	53.64
AES_1663	PA5178	PA14_68400	Putative LysM Domain Protein						3.3535	0.0000	110	69.66
AES_1872	PA1041	PA14_50880	Probable Outer Membrane Protein Precursor		1.4424	0.0090	11	24.76				
AES_0595	PA0291	PA14_03800	Anaerobically-Induced Outer Membrane Porin OprE	*oprE*	0.6919	0.0045	6	11.30	0.4321	0.0296	3	5.83

AES_1776	PA0958	PA14_51880	Basic Amino Acid, Basic Peptide and Imipenem Outer Membrane Porin OprD Precursor	*oprD*	0.4857	0.0064	17	50.56	0.3749	0.0024	17	50.56
AES_4630	PA4067	PA14_11270	Outer Membrane Protein OprG Precursor	*oprG*	0.5498	0.0061	35	42.67	0.6390	0.0004	35	42.67
AES_4157	PA2754	PA14_28490	Putative Membrane Protein		0.5563	0.0031	2	24.77				
AES_6469	PA5022	PA14_66400	Putative Small-Conductance Mechanosensitive Channel Protein		0.6464	0.0006	7	3.58				
AES_5158	PA5231	PA14_69070	Putative ATP-binding/permease fusion ABC transporter		0.6981	0.0095	2	1.20				
AES_0825	PA4208	PA14_09500	Outer Membrane Protein OpmD	*opmD*					0.6828	0.0096	9	19.71
AES_3596	PA2291	PA14_34960	Carbohydrate-Selective Porin	*oprB2*	0.7755	0.0012	7	13.50	0.6980	0.0018	7	13.50
AES_4165	PA2760	PA14_28400	Putative Outer Membrane Porin OprD Family	*oprQ*	0.7250	0.0072	29	32.71	0.6952	0.0099	12	25.88
AES_1788	PA0969	PA14_51750	TolQ Protein	*tolQ*					0.7667	0.0062	11	13.85

DNA Replication and Transcription Proteins

AES_4901	PA3347	PA14_20770	Putative Anti-Sigma F Factor Antagonist		1.6342	0.0132	12	61.39	2.6642	0.0000	39	41.58
AES_5085	PA3618	PA14_17520	Putative Competence-Damaged Protein	*ygaD*	1.7111	0.0258	2	14.88	5.4138	0.0014	2	26.19
AES_4519	PA3940	PA14_12900	Histone-Like Protein HU Form N		1.9729	0.0066	39	58.06	1.5319	0.0125	39	58.06
AES_3988	PA2622	PA14_30200	Cold Shock Protein	*cspD*	2.8091	0.0043	4	63.33				
AES_4411	PA2961	PA14_25760	DNA Polymerase III, Delta Prime Subunit	*holB*	1.7139	0.0056	2	10.67				
AES_5712	PA4238	PA14_09115	DNA-Directed RNA Polymerase Alpha Chain	*rpoA*	1.5763	0.0098	68	68.47				
AES_3334	PA4269	PA14_08780	DNA- Directed RNA Polymerase Beta* Chain	*rpoC*	1.4954	0.0001	137	44.96	1.3547	0.0000	137	44.96
AES_3333	PA4270	PA14_08760	DNA- Directed RNA Polymerase Beta Chain	*rpoB*	1.5378	0.0001	145	50.70	1.3389	0.0000	145	50.70
AES_2395	PA4352	PA14_56590	Putative Universal Stress Protein		1.7650	0.0169	48	65.38	1.4772	0.0475	48	65.38
AES_6178	PA4751	PA14_62860	Cell Division Protein FtsH	*ftsH*	1.7485	0.0088	14	23.16				
AES_4288	PA4764	PA14_63020	Ferric Uptake Regulation Protein Fur	*fur*	1.2744	0.0007	7	41.79	1.5565	0.0088	22	47.01
AES_4665	PA3168	PA14_23260	DNA Gyrase Subunit A	*gyrA*	1.4466	0.0095	18	19.33	1.3171	0.0085	18	19.33
AES_6656	PA5239	PA14_69190	Transcription Termination Factor Rho	*rho*	1.3905	0.0180	26	45.82	1.3474	0.0020	26	45.82
AES_6162	PA4723	PA14_62490	Suppressor Protein DksA	*dksA*	1.4239	0.0100	21	39.86				
AES_6767	PA5339	PA14_70480	Putative Endoribonuclease L-PSP		0.5774	0.0061	7	34.13	0.5731	0.0322	7	34.13

AES_0834	PA0428	PA14_05560	Putative ATP-Dependent RNA Helicase	*rhlE*	0.6687	0.0066	7	11.58				
AES_1780	PA0962	PA14_51830	Probable DNA-Binding Stress Protein		0.6643	0.0000	45	62.18				
AES_4465	PA3004	PA14_25210	Putative Purine Nucleoside Phosphorylase		0.5765	0.0038	8	22.04				
AES_5786	PA4315	PA14_56070	Transcriptional Regulator MvaT P16 subunit	*mvaT*	0.6721	0.0009	25	43.55				
AES_6775	PA5345	PA14_70570	ATP-Dependent DNA Helicase	*recG*	0.5556	0.0045	2	3.62				
AES_4061	PA2667	PA14_29590	Putative Transcriptional Regulator						0.3657	0.0000	53	28.21

Translation, Post-Translational Modification and Chaperones

AES_4660	PA3162	PA14_23330	30S Ribosomal Protein S1	*rpsA*	1.8867	0.0000	106	42.40	1.6610	0.0000	106	42.40
AES_2714	PA3461	PA14_19350	Putative Peptidase or Cellulase	*yhfE*	1.6909	0.0032	3	7.79	1.7569	0.0161	3	7.79
AES_5714	PA4240	PA14_09090	Ribosomal Protein S11	*rpsK*	1.5661	0.0013	17	53.49	1.5863	0.0039	17	53.49
AES_5733	PA4249	PA14_08990	30S Ribosomal Protein S8	*rpsH*	1.9474	0.0279	12	28.46	1.5914	0.0083	12	28.46
AES_5744	PA4273	PA14_08730	50S Ribosomal Protein L1	*rplA*	1.7741	0.0190	56	66.23	1.5133	0.0077	56	66.23
AES_6363	PA4932	PA14_65150	50S Ribosomal Protein L9	*rplI*	2.0347	0.0111	53	54.05	1.8702	0.0084	53	54.05
AES_6366	PA4935	PA14_65180	30S Ribosomal Protein S6	*rpsF*	1.7470	0.0157	19	41.73	1.5947	0.0098	19	41.73
AES_6654	PA5240	PA14_69200	Thioredoxin	*trxA*	2.0798	0.0032	18	56.48	3.0030	0.0000	27	53.70
AES_2963	PA1802	PA14_41230	ATP-Dependent Clp Protease ATP-Binding Subunit	*clpX*	1.6707	0.0033	23	39.20				
AES_5179	PA3700	PA14_16530	Lysyl-tRNA Synthetase	*lysS*	1.6455	0.0495	22	22.55	1.4501	0.0054	20	23.15
AES_1025	PA4176	PA14_09890	Peptidyl-Prolyl Cis-Trans Isomerase C2	*ppiC2*	3.5093	0.0057	3	61.29	1.2731	0.0059	3	61.29
AES_5731	PA4251	PA14_08970	50S Ribosomal Protein L5	*rplE*	1.6143	0.0176	70	58.66	1.4657	0.0021	70	58.66
AES_5719	PA4263	PA14_08850	50S Ribosomal Protein L3	*rplC*	1.7787	0.0087	26	32.70				
AES_3337	PA4266	PA14_08820	Elongation Factor G	*fusA1*	2.5935	0.0000	68	53.54				
AES_5746	PA4271	PA14_08750	50S Ribosomal Protein L7 / L12	*rplL*	1.8005	0.0002	41	89.34				
AES_6099	PA4671	PA14_61780	Ribosomal Protein L25	*rplY*	1.5434	0.0048	51	79.90	1.4807	0.0023	51	79.90
AES_4284	PA4761	PA14_62970	Heat Shock Protein DnaK	*dnaK*	1.5138	0.0048	185	72.84				
AES_2974	PA1803	PA14_41220	Lon Protease	*lon*	1.8003	0.0073	22	27.94	1.1966	0.0263	32	24.81
AES_4262	PA2830	PA14_27480	Heat Shock Protein	*htpX*	1.4063	0.0036	19	26.80	1.5336	0.0363	13	23.71

AES_0345	PA3257	PA14_21880	Periplasmic Tail-Specific Protease	*prc*					2.3236	0.0008	5	7.90
AES_5132	PA3655	PA14_17070	Translation Elongation Factor Ts	*tsf*	1.7247	0.0005	71	62.28	1.2306	0.0355	71	62.28
AES_5724	PA4258	PA14_08900	50S Ribosomal Protein L22	*rplV*					1.5164	0.0036	46	41.82
-	PA4264	PA14_08840	30S Ribosomal Protein S10	*rpsJ*					1.5853	0.0084	9	40.78
AES_5745	PA4272	PA14_08740	50S Ribosomal Protein L10	*rplJ*	1.4309	0.0196	25	43.98	1.4994	0.0020	25	43.98
AES_5843	PA4385	PA14_57010	GroEL Protein	*groEL*					1.5221	0.0066	449	74.22

Metabolic Proteins (Fatty Acid Biosynthesis [FAB] / Amino Acid Biosynthesis / Degradation)

AES_0164	PA0102	PA14_01240	Probable Carbonic Anhydrase		2.4101	0.0167	12	29.34	2.2314	0.0014	12	29.34
AES_1693	PA0895	PA14_52720	N-Succinylglutamate 5-Semialdehyde Dehydrogenase	*aruC*	2.4314	0.0082	11	56.58	1.7107	0.0193	11	56.58
AES_2338	PA1293	PA14_47490	Putative Enzyme		2.5874	0.0002	22	36.26	2.4124	0.0017	22	36.26
AES_2682	PA1589	PA14_43940	Succinyl-CoA Synthetase Alpha Chain	*sucD*	1.7108	0.0025	64	53.90	1.5344	0.0062	64	53.90
AES_2706	PA1609	PA14_43690	Beta-Ketoacyl-ACP Synthase I	*fabB*	2.4873	0.0022	14	36.05	2.0456	0.0006	14	36.05
AES_1056	PA2605	PA14_30400	Putative Sulfur Reductase Protein	*yheN*	2.3356	0.0006	6	22.14	3.1241	0.0198	4	11.45
AES_4015	PA2640	PA14_29980	NADH Dehydrogenase I Chain E	*nuoE*	3.1253	0.0169	4	17.47	2.1069	0.0024	3	12.65
AES_4422	PA2967	PA14_25660	3-Oxoacyl-[Acyl-Carrier Protein] Reductase	*fabG*	3.3974	0.0164	9	32.39	1.7671	0.0056	19	40.49
AES_3765	PA4602	PA14_60890	Glycine/Serine Hydroxymethyltransferase	*glyA3*	2.4338	0.0018	13	15.35	1.5625	0.0181	13	15.35
AES_0239	PA0143	PA14_01760	Non-specific Ribonucleoside Hydrolase	*nuh*	2.0180	0.0061	12	32.83				
AES_0533	PA0265	PA14_03430	Succinate-Semialdehyde Dehydrogenase	*gabD*	1.8451	0.0096	25	47.83	1.4837	0.0007	25	47.83
AES_0679	PA0330	PA14_04310	Ribose 5-Phosphate Isomerase	*rpiA*	1.8103	0.0068	10	32.29				
AES_0927	PA0865	PA14_53070	4-Hydroxyphenylpyruvate Dioxygenase	*hpd*	1.6770	0.0030	68	56.58	0.5355	0.0052	68	56.58
AES_3758	PA2442	PA14_33040	Glycine Cleavage System Protein T2	*gcvT2*	4.0371	0.0088	5	14.75				
AES_4099	PA2709	PA14_29110	Cysteine Synthase A	*cysK*	2.1305	0.0033	5	19.75				
AES_4399	PA2952	PA14_25860	Electron Transfer Flavoprotein Beta Subunit	*etfB*	1.5572	0.0059	28	48.59				
AES_4400	PA2953	PA14_25840	Putative Electron Transfer Flavoprotein-Ubiquinone Oxidoreductase		2.7514	0.0015	18	23.77	1.4502	0.0011	17	21.23
AES_5108	PA3639	PA14_17270	Acetyl-CoA Carboxylase Alpha Subunit	*accA*	1.6853	0.0001	35	50.95	1.4351	0.0002	35	50.95

AES_5588	PA4079	PA14_11130	Putative Short Chain Dehydrogenase		1.5061	0.0083	5	20.52				
AES_1705	PA0904	PA14_52580	Aspartate Kinase Alpha and Beta Chain	*lysC*					2.7753	0.0046	7	10.68
AES_2686	PA1586	PA14_44000	Dihydrolipoamide Succinyltransferase (E2 Subunit)	*sucB*					1.4973	0.0095	73	50.37
AES_2252	PA2069	PA14_37745	Probable Carbamoyl Transferase						2.7935	0.0001	17	22.47
AES_4423	PA2966	PA14_25670	Acyl Carrier Protein	*acpP*					1.9792	0.0002	11	33.33
AES_4608	PA3112	PA14_23860	Acetyl-CoA Carboxylase Beta Subunit	*accD*	1.3467	0.0164	14	23.45	2.0239	0.0000	14	23.45
AES_4653	PA3139	PA14_23500	Putative Amino Acid Aminotransferase	*tyrB; aspC*					1.5712	0.0067	34	46.23
AES_3828	PA4785	PA14_63250	Putative Acyl-CoA Thiolase						1.8985	0.0049	5	17.18
AES_1668	PA5173	PA14_68350	Carbamate Kinase	*arcC*					2.1197	0.0000	23	41.61
AES_1670	PA5171	PA14_68330	Arginine Deiminase	*arcA*	0.6954	0.0383	145	64.83	1.4470	0.0041	214	56.70
AES_1377	PA0745	PA14_54640	Probable Enoyl-CoA Hydratase/Isomerase		0.6004	0.0000	30	46.32	0.7873	0.0345	30	46.32
AES_3543	PA2250	PA14_35490	Lipoamide Dehydrogenase-Val	*lpdV*	0.5345	0.0000	125	62.07	0.7992	0.0051	125	62.07
AES_5105	PA3636	PA14_17310	2-Dehydro-3-Deoxyphosphooctonate Aldolase	*kdsA*	0.5370	0.0068	18	17.08	0.7550	0.0175	6	16.01
AES_0346	PA3256	PA14_21890	Putative Quinone Oxidoreductase		0.6759	0.0023	8	9.69				
AES_0073	PA3366	PA14_20560	Aliphatic Amidase	*amiE*	0.5070	0.0143	6	8.67				
AES_4991	PA3996	PA14_12130	Lipoate Synthase	*lis*	0.6038	0.0004	3	5.20	0.8744	0.0259	3	5.20
AES_5901	PA4431	PA14_57570	Putative Cytochrome c Reductase, Iron-Sulfur Subunit		0.5823	0.0011	17	32.99				
AES_1669	PA5172	PA14_68340	Ornithine Carbamoyltransferase	*arcB*	0.5944	0.0002	242	58.33	1.4206	0.0000	82	59.52
AES_1656	PA5184	PA14_68480	Putative Periplasmic Chorismate Mutase		0.6639	0.0032	3	23.24				
AES_6914	PA5553	PA14_73230	ATP Synthase Epsilon Chain	*atpC*	0.6453	0.0214	8	48.23				
AES_0869	PA0446	PA14_05820	Putative Acyl-CoA Transferase						0.5939	0.0091	6	10.81
AES_0204	PA0747	PA14_54620	Probable Aldehyde Dehydrogenase						0.6337	0.0063	4	9.96
AES_3906	PA2540	PA14_31720	Putative Lysophospholipase						0.6573	0.0008	4	4.95
AES_5849	PA4389	PA14_57050	Probable Short-Chain Dehydrogenase	*speA*					0.5798	0.0029	6	17.86

Hypothetical proteins

AES_7165	-	-	Hypothetical Protein		4.1526	0.0308	5	24.07	4.8960	0.0013	3	24.07
AES_0637	PA0315	PA14_04100	Conserved Hypothetical Protein		1.5228	0.0041	10	36.55	2.5466	0.0207	5	36.55
AES_1536	PA0833	PA14_53500	Hypothetical Protein		1.6175	0.0256	13	46.41	3.1256	0.0002	6	41.35
AES_5488	PA4005	PA14_12030	Conserved Hypothetical Protein		2.3688	0.0050	5	23.73	1.6222	0.0007	5	23.73
AES_0883	PA0460	PA14_06010	Conserved Hypothetical Protein		1.8659	0.0063	14	41.15	1.2685	0.0447	14	41.15
AES_6165	PA4735	PA14_62650	Conserved Hypothetical Protein		1.7217	0.0044	9	6.89	1.3444	0.0412	9	6.89
AES_6169	PA4738	PA14_62680	Conserved Hypothetical Protein		1.5007	0.0020	27	33.85	0.7070	0.0378	16	33.85
AES_0680	PA0329	PA14_04300	Conserved Hypothetical Protein						2.4904	0.0004	16	37.07
AES_3524	PA2235	PA14_35690	Hypothetical Protein	*pslE*					1.6654	0.0272	26	18.13
AES_5515	PA4015	PA14_11910	(R)-Specfic Enoyl-CoA Hydratase	*phaJ4*					2.0609	0.0000	15	51.66
AES_5858	PA4395	PA14_57130	Conserved Hypothetical Protein						1.5785	0.0089	20	42.77
AES_3210	PA4441	PA14_57690	Conserved Hypothetical Protein						2.8918	0.0001	13	47.97
AES_1176	PA0565	PA14_07355	Hypothetical Protein		0.6567	0.0002	7	18.75				
AES_1757	PA0943	PA14_52060	Conserved Hypothetical Protein		0.5491	0.0063	6	6.30				
AES_2192	PA1216	PA14_48590	Conserved Hypothetical Protein		0.6678	0.0038	5	17.74				
AES_3134	PA1913	PA14_39790	Conserved Hypothetical Protein		0.4476	0.0062	2	10.62				
AES_5217	PA3729	PA14_16180	Conserved Hypothetical Protein		0.6722	0.0014	16	17.49				
AES_6297	PA5494	PA14_72500	Conserved Hypothetical Protein		0.5483	0.0004	5	9.38	1.3341	0.0121	3	9.38
-	-	PA14_53590	Hypothetical Protein						0.1846	0.0092	3	4.49
AES_0804	PA0418	PA14_05440	Hypothetical Protein						0.6617	0.0100	3	5.94
AES_2373	PA1324	PA14_47120	Hypothetical Protein						0.5786	0.0004	9	31.18
AES_1751	PA0938	PA14_52130	Hypothetical Protein						0.6989	0.0005	17	17.65
AES_0226	PA0141	PA14_01730	Conserved Hypothetical Protein		0.7398	0.0084	9	28.19				28.19

Proteins were identified by 2-DLC-MS/MS and quantitated by iTRAQ labelling. AES No., PAO1 No. and PA14 No. refer to translated ORF number from AES-1, PAO1 and PA14 genome sequence. No. Pept. refers to number of identified and iTRAQ labeled peptides used to perform quantitation and statistical analysis; Seq. Cov. (%), % of the sequence covered by matching peptides; AES-1R v PA14 (PAO1), *n*-fold difference

in abundance in AES-1R compared to PA14 and PAO1. Proteins with a ratio >1.5 (shaded red; p<0.05, shaded light green) or <0.67 (shaded blue; p<0.05), or >1.3 (also shaded red; p<0.01, shaded dark green) or <0.77 (p<0.01; also shaded blue) were considered to be statistically significantly altered in abundance. Proteins meeting the fold change criteria but not the accepted p-values are shaded in yellow. Proteins not meeting the fold criteria but for which acceptable p-values were obtained (providing statistical evidence of a small fold change) are not shaded.
