# Supplementary material for: Proteomic profiling of Pseudomonas aeruginosa AES-1R, PAO1 and PA14 reveals potential virulence determinants associated with a transmissible cystic fibrosis-associated strain
Source: BMC Microbiol. 2012 Jan 22;12:16. doi: 10.1186/1471-2180-12-16 (PMC3398322; doi:10.1186/1471-2180-12-16)
Supplement: Additional file 4 — Figure showing overlap of identified and quantified proteins by 2-DE and 2-DLC/MS with iTRAQ. Table showing relative abundance changes for 22 proteins quantified by both 2-DE and iTRAQ. [file 1471-2180-12-16-S4.PDF]

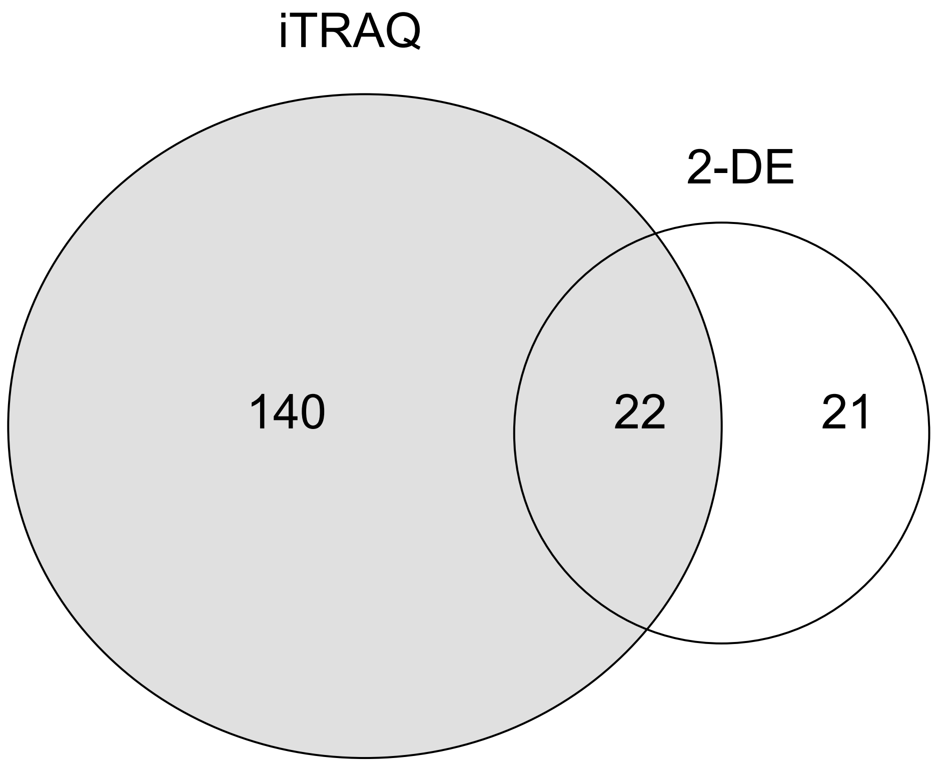
iTRAQ
2-DE
140
22
21

2-DE Spot No.	AES No.	PAO1 No.	PA14 No.	Protein Name	Gene Name	iTRAQ AES-1R v PA14	p-value	iTRAQ AES-1R v PAO1	p-value	2-DE AES-1R v PA14	p-value	2-DE AES-1R v PAO1	p-value
2	AES_5685	PA4221	PA14_09340	Fe(III)-Pyochelin Outer Membrane Receptor FptA	*fptA*	0.57	0.0003		n.s	0.66	0.037	0.82	0.245
4	AES_4284	PA4761	PA14_62970	Heat Shock Protein DnaK	*dnaK*		n.s	1.51	0.0048	+	+	2.52	0.003
5	AES_4660	PA3162	PA14_23330	30S Ribosomal Protein S1	*rpsA*	1.66	0.0000	1.89	0.0000	+	+	1.52	0.002
9a *	AES_3607	PA2300	PA14_34870	Chitinase ChiC	*chiC*		n.s	1.52	0.0040	1.88	0.001	1.42	0.001
10a *	AES_5843	PA4385	PA14_57010	GroEL protein	*groEL*	1.52	0.0066		n.s	+	+	2.37	0.002
11	AES_3596	PA2291	PA14_34960	Carbohydrate-Selective Porin	*oprB2*	0.70	0.0018	0.77	0.0012	1.00	0.984	0.73	0.046
12	AES_0595	PA0291	PA14_03800	Anaerobically-Induced Outer Membrane Porin OprE	*oprE*	0.43	0.0296	0.69	0.0045	0.80	0.010	0.73	0.004
15	AES_1776	PA0958	PA14_51880	Basic Amino Acid, Basic Peptide and Imipenem Outer Membrane Porin OprD Precursor	*oprD*	0.37	0.0024	0.49	0.0064	-	-	-	-
16	AES_4165	PA2760	PA14_28400	Putative Outer Membrane Porin OprD Family	*oprQ*	0.70	0.0099	0.72	0.0072	1.50	0.038	1.06	0.637
17	AES_1670	PA5171	PA14_68330	Arginine Deiminase	*arcA*	1.45	0.0041	0.70	0.0383	3.05	0.047	1.06	0.284
18	AES_0447	PA4217	PA14_09400	Flavin-Containing Monooxygenase PhzS	*phzS*		n.s	1.47	0.0059	+	+	0.81	0.304
20a *	AES_1669	PA5172	PA14_68340	Ornithine Carbamoyltransferase	*arcB*	1.42	0.0000	0.59	0.0002	0.93	0.377	1.86	0.016
23	AES_6636	PA5217	PA14_68900	Putative Iron ABC Transporter, Periplasmic Iron-Binding Protein		1.63	0.0110	1.79	0.0013	+	+	+	+
24	AES_1668	PA5173	PA14_68350	Carbamate Kinase	*arcC*	2.12	0.0000		n.s.	+	+	1.33	0.041
25	AES_2395	PA4352	PA14_56590	Putative Universal Stress Protein		1.48	0.0475	1.77	0.0169	1.58	0.001	1.22	0.175
27	AES_5171	PA3692	PA14_16630	Putative Outer Membrane Protein (Fragment)	*ompA*	2.32	0.0000	3.98	0.0006	1.43	0.028	1.04	0.784
28	AES_4630	PA4067	PA14_11270	Outer Membrane Protein OprG Precursor	*oprG*	0.64	0.0004	0.55	0.0061	0.48	0.000	0.56	0.004
35	AES_6067	PA4880	PA14_64520	Putative Bacterioferritin			n.s	3.40	0.0034	+	+	1.15	0.358
36	AES_0365	PA2331	PA14_34460	Putative Alkylhydroperoxidase		2.00	0.0004		n.s	+	+	0.82	0.315
38	AES_6094	PA4661	PA14_61650	Lipid A 3-O-Deacylase	*pagL*	0.60	0.0000		n.s	-	-	-	-
40	AES_1663	PA5178	PA14_68400	Putative LysM Domain Protein		3.35	0.0000		n.s	+	+	1.47	0.004
42	AES_5710	PA4236	PA14_09150	Catalase KatA	*katA*		n.s	0.32	0.0037	0.53	0.038	0.46	0.029

Additional File 4. Upper: Venn diagram showing comparison of 2-DE and iTRAQ 2-DLC/MS-MS for quantified proteins changing in abundance between *P. aeruginosa* AES-1R, PAO1 and PA14. **Lower:** Comparison of quantitative differences detected between AES-1R and PAO1 or PA14 by gel-based (2-DE) and gel-free (iTRAQ 2-DLC/MS-MS) proteomics approaches for 22 proteins identified as changing in abundance by both methods. 2-DE-separated proteins were identified by peptide mass mapping and quantitated by densitometry of 2-DE gels; 2-DLC/MS-MS identified proteins were quantitated by iTRAQ labeling. Spot No. refers to spot number from 2-DE gels (Figure 1). AES No., PAO1 No. and PA14 No. refer to translated ORF number from AES-1R, PAO1 and PA14 genome sequence. AES-1R v PA14 (PAO1), *n*-fold difference in abundance in AES-1R compared to PA14 and PAO1 (see Additional File 2 and Additional File 3). Proteins elevated in abundance in AES-1R compared to PAO1 or PA14 are shaded red. Proteins reduced in abundance in AES-1R are shaded blue. Significant p-values are shaded green. +, Spot only detected in AES-1R; -, Spot not detected in AES-1R. n.s; protein identified but iTRAQ ratio did not reach statistical significance. *, protein characterized by more than one spot changing in abundance on 2-DE gels (see Additional File 2).
