# Supplementary material for: Proteomic profiling of Pseudomonas aeruginosa AES-1R, PAO1 and PA14 reveals potential virulence determinants associated with a transmissible cystic fibrosis-associated strain
Source: BMC Microbiol. 2012 Jan 22;12:16. doi: 10.1186/1471-2180-12-16 (PMC3398322; doi:10.1186/1471-2180-12-16)
Supplement: Additional file 5 — Protein sequence alignment of Flagellin (FliC/FlaA) of P. aeruginosa strains used in this study (AES_1954, PA1092, and PA14_50290) and including an additional sequence from strain PAK with a known type A flagellin. The flagellin sequence of strain AES-1R has higher sequence similarity with the shorter A type flagellin of strain PAK (95%), while the type B flagellins of strains PA14 and PAO1 are almost identical with only a single amino acid difference. [file 1471-2180-12-16-S5.PDF]

Additional File 5: Protein sequence alignment of Flagellin (FliC/FlaA) of *P. aeruginosa* strains used in this study (AES_1954, PA1092, and PA14_50290) and including an additional sequence from strain PAK with a known type A flagellin. The flagellin sequence of strain AES-1R has higher sequence similarity with the shorter A type flagellin of strain PAK (95%), while the type B flagellins of strains PA14 and PAO1 are almost identical with only a single amino acid difference.

```
AES_1954            MALTVNTNIASLNTQRNLNNSSASLNTSLQRLSTGSRINSAKDDAAGLQIANRLTSQVNG 60
PAK FlaA            MALTVNTNIASLNTQRNLNNSSASLNTSLQRLSTGSRINSAKDDAAGLQIANRLTSQVNG 60
PA1092              MALTVNTNIASLNTQRNLNASSNDLNTSLQRLTTGYRINSAKDDAAGLQISNRLSNQISG 60
PA14_50290          MALTVNTNIASLNTQRNLNASSNDLNTSLQRLTTGYRINSAKDDAAGLQISNRLSNQISG 60
                    ******************* ** .********:** **************:***:.*:.*

AES_1954            LNVATKNANDGISLAQTAEGALQQSTNILQRMRDLSLQSANGSNSDSERTALNGEVKQLQ 120
PAK                 LNVATKNANDGISLAQTAEGALQQSTNILQRMRDLSLQSANGSNSDSERTALNGEAKQLQ 120
PA1092              LNVATRNANDGISLAQTAEGALQQSTNILQRIRDLALQSANGSNSDADRAALQKEVAAQQ 120
PA14_50290          LNVATRNANDGISLAQTAEGALQQSTNILQRIRDLALQSANGSNSDADRAALQKEVAAQQ 120
                    *****:*************************:***:**********::*:**: *.   *

AES_1954            KELDRISNTTTFGGRKLLDGSFGVASFQVGSAANEIISVGIDEMSAESLNGTYFKADGGG 180
PAK                 KELDRISNTTTFGGRKLLDGSFGVASFQVGSAANEIISVGIDEMSAESLNGTYFKADGGG 180
PA1092              AELTRISDTTTFGGRKLLDGSFGTTSFQVGSNAYETIDISLQNASASAIGSYQVGSNGAG 180
PA14_50290          AELTRISDTTTFGGRKLLDGSFGTTSFQVGSNAYETIDISLQNASASAIGSYQVGSNGAG 180
                     ** ***:***************.:****** * * *.:.::: **.::..  . ::*.*

AES_1954            AVTAAT--------ASGTVDIAIG------------------------------------ 196
PAK                 AVTAAT--------ASGTVDIAIG------------------------------------ 196
PA1092              TVASVAGTATASGIASGTVNLVGGGQVKNIAIAAGDSAKAIAEKMDGAIPNLSARARTVF 240
PA14_50290          TVASVAGTATASGIASGTVNLVGGGQVKNIAIAAGDSAKAIAEKMDGAIPNLSARARTVF 240
                    :*::.:        *****::. *

AES_1954            ------ITGG---------------SAVNVKVDMKGNETAEQAAAKIAAAVND------- 228
PAK                 ------ITGG---------------SAVNVKVDMKGNETAEQAAAKIAAAVND------- 228
PA1092              TADVSGVTGGSLNFDVTVGSNTVSLAGVTSTQDLADQLNSNSSKLGITASINDKGVLTIT 300
PA14_50290          TADVSGVTGGSLNFDVTVGSNTVSLAGVTSTQDLADQLNSNSSKLGITASINDKGVLTIT 300
                          :***               :.*. . *: .: .::.:   *:*::**

AES_1954            ----ANVGIGAFT---------------DGAQISYVSKASADGT---TSAVSGVAITDT- 265
PAK                 ----ANVGIGAFS---------------DGDTISYVSKAGKDGSGAITSAVSGVVIADT- 268
PA1092              SATGENVKFGAQTGTATAGQVAVKVQGSDGKFEAAAKNVVAAGTAATTTIVTGYVQLNSP 360
PA14_50290          SATGENVKFGAQTGTATAGQVAVKVQGSDGKFEAAAKNVVAAGTAATTTVVTGYVQLNSP 360
                         ** :** :               **   : ..:.   *:   *: *:* .  ::

AES_1954            ---GSTGAGTAAG----TTTFTEANDTVAKIDISTAKGAQSAVLVIDEAIKQIDAQRADL 318
PAK                 ---GSTGVGTAAGVAPSATAFAKTNDTVAKIDISTAKALSRRAGDRTTAIKQIDASVPTS 325
PA1092              TAYSVSGTGTQAS-QVFGNASAAQKSSVASVDISTADGAQNAIAVVDNALAAIDAQRADL 419
PA14_50290          TAYSVSGTGTQAS-QVFGNASAAQKSSVASVDISTADGAQNAIAVVDNALAAIDAQRADL 419
                       . :*.** *.     .: :  :.:**.:*****.. .        *:  ***. .

AES_1954            GAVQNRFDNTINNLKNIGENVSAARGRIEDTDFAAETANLTKNQVLQQAGTAILAQANQL 378
PAK                 VAVQNRFDNTINNLKNIGENVSAARGRIEDTDFAAETANLTKNQVLQQAGTAILAQANQL 385
PA1092              GAVQNRFKNTIDNLTNISENATNARSRIKDTDFAAETAALSKNQVLQQAGTAILAQANQL 479
PA14_50290          GAVQNRFKNTIDNLTNISENATNARSRIKDTDFAAETAALSKNQVLQQAGTAILAQANQL 479
                     ******.***:**.**.**.: **.**:********* *:*******************

AES_1954            PQSVLSLLR 387
PAK                 PQSVLSLLR 394
PA1092              PQAVLSLLR 488
PA14_50290          PQAVLSLLR 488
                    **:******
```
